# Second-order neutrosophic boundary-value problem

**DOI:** 10.1007/s40747-020-00268-8

**Published:** 2021-01-24

**Authors:** Sandip Moi, Suvankar Biswas, Smita Pal(Sarkar)

**Affiliations:** 1 Department of Mathematics, Sonarpur Mahavidyalaya, Kolkata, 700149 India; 2 Department of Mathematics, Indian Institute of Engineering Science and Technology, Shibpur, B. Garden, Howrah, 711103 India

**Keywords:** Neutrosophic differential calculus, Second-order neutrosophic differential equation, Neutrosophic boundary-value problem, Generalized neutrosophic derivative

## Abstract

In this article, some properties of neutrosophic derivative and neutrosophic numbers have been presented. This properties have been used to develop the neutrosophic differential calculus. By considering different types of first- and second-order derivatives, different kind of systems of derivatives have been developed. This is the first time where a second-order neutrosophic boundary-value problem has been introduced with different types of first- and second-order derivatives. Some numerical examples have been examined to explain different systems of neutrosophic differential equation.

## Introduction

In 1965, Lotfi A Zadeh [1, 2] introduced fuzzy set theory. After that, there are various kinds of generalizations of fuzzy sets which have been introduced by many researchers [3–6]. Neutrosophic set is one of them. Smarandache [5–7] gives the concept of neutrosophic set theory to explain more complex system than fuzzy where the falsity-membership value is not the complement of truth membership value and an indeterminacy in play about the assignment of values of truth membership and falsity-membership function. After the invention of neutrosophic set, a new branch came in the field of fuzzy mathematics, which needs further development of the different fields of neutrosophic mathematics like Neutrosophic Vector Space [8], Neutrosophic Topological Space [9], Neutrosophic Group Theory [10], Neutrosophic Ring Theory [11], and Neutrosophic Differential Equation [12, 13], etc. In the recent time, many researchers are still working on the development of neutrosophic set theory and its various types of applications. Topal et al. [14] used neutrosophic environment to construct Bezier surface modeling for data problems. In [15], Broumi et al. introduced the uniform single-valued neutrosophic graph and they also develop an algorithm to compute the complement of single-valued neutrosophic graph. Also Broumi et al. [16] applied neutrosophic set theory in some computing procedures in Matlab for operational matrices. After that, Broumi et al. [17–19] used neutrosophic environment to solve some shortest path problems. Saranya et al. [20] proposed a computer-based application, which helps to find the values of union, intersection, compliment, and inclusion of any two neutrosophic set. Gulistan et al. [21] extended the concept of neutrosophic cubic sets with the help of neutrosophic sets, cubic sets, and complex fuzzy sets. Du et al. [22] introduced neutrosophic Z-number and their operations. Aslam [23] used neutrosophic statistical interval method to introduced a new sampling plan. Edalatpanah [24] proposed a new algorithm to solve the neutrosophic linear programming, where the variables were taken as triangular neutrosophic number. Recently, Salama et al. [25] proposed a diagnostic system of corona virus which is based on the neutrosophic system.

### Neutrosophic calculus

In our literature review, we have seen that the neutrosophic precalculus and neutrosophic calculus were first studied by Smarandache [26], which is based on the existing definition of calculus. Neutrosophic derivative was first introduced by Smarandache [26]. Neutrosophic derivative is the extension of fuzzy derivative. The granular derivative(gr-derivative) is a new type of neutrosophic derivative, which was introduced by Son et al. [27]. Also, Son et al. [27] gave the gr-partial derivative of neutrosophic-valued several variable function. The neutrosophic granular fractional derivative was also given by Son et al. [27]. Again Son et al. investigated the if and only if condition for the existence of gr-derivative of neutrosophic-valued function.

Neutrosophic integral calculus plays very important role in the field of neutrosophic calculus. Neutrosophic integral is the extension of fuzzy integral, which was first introduced by Smarandache [26]. In the article [28], they have studied the neutrosophic calculus using measure theory and neutrosophic probability theory. In the recent time, there are only few works which have been done on neutrosophic integral calculus. Therefore, there are lots of scope to develop the neutrosophic integral calculus.

### Neutrosophic differential equation

Before discussing about neutrosophic differential equation, we should know about fuzzy differential equation which may be modified or generalized for neutrosophic environment. The theory of fuzzy differential equation was first introduced by Kaleva [29], which had been developed in the form of Hukuhara derivative. After that there are various types of work on fuzzy differential equation which have been studied by different researchers and various types of work on this field are still going on. Some of these important work, which may help to develop the work on neutrosophic differential equation, have been listed here. Seikkala [30] introduced fuzzy initial value problem, where he applied extension principle and extremal solutions of deterministic initial value problems to solve the fuzzy differential equation. Then, Bede et al. [31] introduced strong generalized differentiability and weak generalized differentiability; with the help of this generalized differentiability, they have obtained the solution of fuzzy differential and partial differential equation. Lakshmikantham et al. [32] studied the conditions for the existence and uniqueness of the solution of boundary-value problem in fuzzy environment. Then, Lakshmikantham et al. [32] and O’Regan et al. [33] show that the second-order fuzzy boundary-value problem is equivalent to Fredholm integral equation. However, Bede [34] prove that there assertion does not true by a counter example. Ma et al. [35] introduced a numerical technique based on classical Euler method to solve fuzzy differential equation. Then, Abbasbandy et al. [36] presented an another numerical technique based on Taylor Method of order p to solve fuzzy differential equation. After that, Bede [37] proposed the characterization theorems to solve the fuzzy differential equation. Khastan et al. [38] introduced a new concept to solve fuzzy boundary-value problem using a generalized differentiability where they investigate the problem to find solutions in different (n,m)-system, where $n,m\in \{1,2\}$. Tapaswini et al. [39] proposed polynomial collocation method to solve fuzzy differential equation. In the recent time, Balakrishnan et al. [40] studied the fifth-order Milne–Simpson method to find the solution of fuzzy differential equation using interval-valued fuzzy number. There are many researchers, who are still working for analytical and numerical solution of fuzzy differential equation [41–43]. Now, all the above work may be modified and generalized for neutrosophic environment.

In the recent time, researcher is also working on neutrosophic differential equation. Sumanthi et al. [12] proposed a method to solve differential equation involving neutrosophic numbers with an application in the field of bacteria culture model. Thereafter, Sumanthi et al. [13] discuss about the solution a neutrosophic differential equation where they have taken trapezoidal neutrosophic number as boundary conditions. Recently, Son et al. [27] introduced some derivatives in the form of fractional order and they also introduced the concept of neutrosophic derivatives in fractional calculus.

### Motivation

In our literature review, we have been seen that there are few works have been done on neutrosophic differential equation. However, there are almost no work have been done on neutrosophic boundary-value problem and neutrosophic initial value problem. Therefore, there is a big scope and opportunity to work in these area. Since there is not much work which have been done, then we must develop the basic properties and results which are needed for the proper development of this topic. Now, a proper development of basic differential equation already have been done in crisp and fuzzy environment which motivates us to think about the similar types of development and modification in neutrosophic environment. In the future, this article may help the other researcher for the further development of this topic.

### Novelty

To build a theory of neutrosophic differential equation, the second-order neutrosophic boundary-value problem is developed in this article.

The objective of this article are presented as follows :

- To present some properties of neutrosophic number.
- To prove the neutrosophic derivative [26] and generalized neutrosophic derivative [44] are equivalent.
- To present (*n*, *m*)-types differentiability of neutrosophic-valued function.
- To prove subtraction of two first-order and second-order neutrosophic differentiable function is also differentiable.
- To prove multiplication of two neutrosophic differentiable function is also differentiable.
- To define two-point neutrosophic boundary-value problem in different (*n*, *m*) system, where $n,m\in \{1,2\}$.
- To solve two-point neutrosophic boundary-value problem and investigate the solutions in different (*n*, *m*)-system.

In this article, we are going to develop the theory of neutrosophic differential equation. To do that we are going to develop some properties of neutrosophic number in the form of some propositions. We are going to present some theorems on neutrosophic derivative, which will help us to develop this article.

### Structure of the paper

The article has been organized as follows: some mathematical preliminaries have been given in Sect. 2, which is related to our article. Section 3 contains some properties of neutrosophic number, definitions, and propositions. In Sect. 4, generalized neutrosophic derivative has been given in the form of some definitions and theorems. The neutrosophic boundary-value problem has been defined in Sect. 5. Some test examples have been investigated in Sect. 6. Finally, a brief conclusion about this article has been given in Sect 7.

## Preliminaries

### Definition 2.1

[45] An single-valued neutrosophic set (SVN-set) N over the universal set U is a neutrosophic set over U, but the truth, indeterminacy, and falsity-membership function are, respectively, defined by $T_{N} : U \rightarrow [0, 1]$,   $I_{N} : U \rightarrow [0, 1]$,   $F_{N} : U \rightarrow [0, 1]$.

### Definition 2.2

[13] A neutrosophic set N over the set of real numbers ${\mathbb {R}}$ is said to be neutrosophic number if its satisfy the following properties.

1. N is normal ie., there exists $x_0\in {\mathbb {R}}$, such that $T_N(x_0)=1$.($I_N(x_0)=F_N(x_0)=0$).
2. N is convex for truth function $T_N(x)$, i.e., $T_N(\mu x_1+(1-\mu )x_2)\ge min(T_N(x_1),T_N(x_2))$,$\forall x_1,x_2 \in {\mathbb {R}}$, and $\mu \in [0,1]$.
3. N is concave for indeterministic and falsity functions, $I_N(x)$ and $F_N(x)$,, respectively, i.e., $I_N(\mu x_1+(1-\mu )x_2)\ge max(I_N(x_1),I_N(x_2))$, and $F_N(\mu x_1+(1-\mu )x_2)\ge max(F_N(x_1),F_N(x_2))$ $\forall x_1,x_2 \in {\mathbb {R}}$ and $\mu \in [0,1]$.

### Definition 2.3

[45] A single-valued triangular neutrosophic number (SVTN-number) $N=\langle (p,q,r); \rho _N, \nu _N, \kappa _N \rangle$ is a special neutrosophic set on ${\mathbb {R}}$, whose truth, indeterminacy, and falsity-membership functions are defined by:

$$\begin{aligned}&T_{N}(x)= {\left\{ \begin{array}{ll} \left( \dfrac{x-p}{q-p}\right) \rho _{N} &{} \text {for}\ p\le x \le q \\ \left( \dfrac{r-x}{r-q}\right) \rho _{N} &{} \text {for}\ q\le x \le r \\ ~~~~0 &{} \text {Otherwise} \end{array}\right. }\\&I_{N}(x)= {\left\{ \begin{array}{ll} \dfrac{(q-x+\nu _N(x-p))}{q-p} &{} \text {for}\ p\le x \le q \\ \dfrac{(x-q+\nu _N(r-x))}{r-q} &{} \text {for}\ q\le x \le r \\ ~~~~~~~~~~~0 &{} \text {Otherwise} \end{array}\right. }\\&F_{N}(x)= {\left\{ \begin{array}{ll} \dfrac{(q-x+\kappa _N(x-p))}{q-p}&{} \text {for}\ p\le x \le q \\ \dfrac{(x-q+\kappa _N(r-x))}{r-q} &{} \text {for}\ q\le x \le r \\ ~~~~~~~~~~~0 &{} \text {Otherwise}. \end{array}\right. } \end{aligned}$$

### Definition 2.4

[45] A single-valued trapezoidal neutrosophic number (SVTrN-number) $N=\langle (p,q,r,s); \rho _N, \nu _N, \kappa _N \rangle$ is a special neutrosophic set on ${\mathbb {R}}$, whose truth, indeterminacy, and falsity-membership functions are defined by:

$$\begin{aligned}&T_{N}(x)= {\left\{ \begin{array}{ll} \left( \dfrac{x-p}{q-p}\right) \rho _{N} &{} \text {for}\ p\le x \le q \\ \quad \rho _{N} &{} \text {for}\ q\le x \le r \\ \left( \dfrac{s-x}{s-r}\right) \rho _{N} &{} \text {for}\ r \le x \le s \\ \quad 0 &{} \text {Otherwise} \end{array}\right. }\\&I_{N}(x)= {\left\{ \begin{array}{ll} \dfrac{(q-x+\nu _N(x-p))}{q-p} &{} \text {for}\ p\le x \le q \\ ~~~~~~~~~~\nu _{N} &{} \text {for}\ q\le x \le r \\ \dfrac{(x-r+\nu _N(s-x))}{s-r}&{} \text {for}\ r \le x \le s \\ ~~~~~~~~~~~0 &{} \text {Otherwise} \end{array}\right. }\\&F_{N}(x)= {\left\{ \begin{array}{ll} \dfrac{(q-x+\kappa _N(x-p))}{q-p} &{} \text {for}\ p\le x \le q \\ \qquad \kappa _{N} &{} \text {for}\ q\le x \le r \\ \dfrac{(x-r+\kappa _N(s-x))}{s-r}&{} \text {for}\ r \le x \le s \\ \qquad 0 &{} \text {Otherwise}. \end{array}\right. } \end{aligned}$$

### Definition 2.5

[13] Let N be a neutrosophic set. Then, $(\alpha ,\beta ,\gamma )$-cut of N is denoted by $N_{(\alpha ,\beta ,\gamma )}$, where $\alpha ,\beta ,\gamma \in [0,1]$, such that $N_{(\alpha ,\beta ,\gamma )}=\{\langle T_N(x),I_N(x),F_N(x)\rangle : x\in U, T_N(x)\ge \alpha , I_N(x)\le \beta , F_N(x)\le \gamma \}$.

### Definition 2.6

[26] The neutrosophic derivative of the neutrosophic-valued function $f_{Neu}(X)$ is defined by:

$$\begin{aligned}&f'_{Neu}(X)= \displaystyle \lim _{\sigma (H) \rightarrow 0}\\&\quad \dfrac{\langle inf f(X+H)-inf f(X), sup f(X+H)- sup f(X)\rangle }{H}, \end{aligned}$$

where $\langle a,b \rangle$ denote the open/closed/half open-closed interval and $\sigma (H)$=max$\{|inf H|, |sup H|\}$

When H is an interval, then the definition written as:

$$\begin{aligned}&f'_{Neu}(X)= \displaystyle \lim _{[inf H, sup H] \rightarrow [0, 0]}\\&\quad \dfrac{[inf f(X+H)-inf f(X), sup f(X+H)-sup f(X)]}{[inf H, sup H]} \end{aligned}$$

is neutrosophic derivative of the function *f*(*X*).

Then, it can be written as:

$$\begin{aligned}&f'_{Neu}(X)= \displaystyle \lim _{h \rightarrow 0}\\&\quad \dfrac{[inf f(X+H)-inf f(X), sup f(X+H)-sup f(X)]}{h}. \end{aligned}$$

Both definitions are the generalizations of the classical derivative of a function, and then, for the crisp functions and for the crisp variables, we have:

$$[inf H, sup H]\equiv h$$ and $$inf f(X+H)\equiv sup f(X+H) \equiv f(x+h)$$, $$inf f(X)\equiv sup f(X)\equiv f(x)$$

## Some properties of neutrosophic number

### Definition 3.1

[46] Let ${\tilde{A}}$ and ${\tilde{B}}$ be two single-valued neutrosophic set on $X=\{x_1,x_2,\ldots ,x_n\}$. Then, the Hausdorff distance measure between ${\tilde{A}}$ and ${\tilde{B}}$ on X is:

$$\begin{aligned}&D^N_{Hau}({\tilde{A}},{\tilde{B}})=\dfrac{1}{n}\sum _{i=1}^n\max \\&\quad \{|T_{{\tilde{A}}}(x_i)\!-\!T_{{\tilde{B}}}(x_i)|,|I_{{\tilde{A}}}(x_i)-I_{{\tilde{B}}}(x_i)|,|F_{{\tilde{A}}}(x_i)\!-\!F_{{\tilde{B}}}(x_i)| \}. \end{aligned}$$

### Proposition 3.1

Let ${\tilde{m}}$ and ${\tilde{n}}$ be the two neutrosophic numbers, and then:

1. $({\tilde{m}}\odot {\tilde{n}})_{(\alpha ,\beta ,\gamma )}={\tilde{m}}_{(\alpha ,\beta ,\gamma )}\odot {\tilde{n}}_{(\alpha ,\beta ,\gamma )}$, where $\odot$ denotes any binary operation $'+','-'$ and $'\times '$.
2. $(\lambda {\tilde{m}})_{(\alpha ,\beta ,\gamma )}=\lambda {\tilde{m}}_{(\alpha ,\beta ,\gamma )}$, where $\lambda \ne 0$ be any real number.

### Proof

Proof of this proposition is trivial and it can be done using extension principle [47]. $\square$

### Proposition 3.2

Let $a,b,\lambda \in {\mathbb {R}}$, $a,b\ge 0$ or $a,b\le 0$ and ${\tilde{m}}, {\tilde{n}} \in {\mathcal {N}}$, where ${\mathcal {N}}$ is the set of all neutrosophic number, and then:

1. $$(a+b){\tilde{m}}=a{\tilde{m}}+b{\tilde{m}}$$
2. $$\lambda ({\tilde{m}}+{\tilde{n}})=\lambda {\tilde{m}}+\lambda {\tilde{n}}$$.

### Proof

1. Since $a,b\ge 0$, this implies that $a+b\ge 0$ Then, by Proposition 3.1:
  $$\begin{aligned}&{[}(a+b){\tilde{m}}]_{(\alpha ,\beta ,\gamma )}=(a+b){\tilde{m}}_{(\alpha ,\beta ,\gamma )}\\&\quad =(a+b)\langle [m_{\alpha }^L,m_{\alpha }^U],\\&\qquad [m_{\beta }^L,m_{\beta }^U],[m_{\gamma }^L,m_{\gamma }^U]\rangle \\&{[}(a+b){\tilde{m}}]_{(\alpha ,\beta ,\gamma )}\\&\quad =(a+b)\langle [m_{\alpha }^L,m_{\alpha }^U],[m_{\beta }^L,m_{\beta }^U],\\&\qquad [m_{\gamma }^L,m_{\gamma }^U]\rangle \\&\quad =\langle [(a+b)m_{\alpha }^L,(a+b)m_{\alpha }^U],[(a+b)m_{\beta }^L,\\&\qquad (a+b)m_{\beta }^U],[(a+b)m_{\gamma }^L,(a+b)m_{\gamma }^U]\rangle \\&\quad =\langle [am_{\alpha }^L+bm_{\alpha }^L,am_{\alpha }^U+bm_{\alpha }^U],\\&\qquad [am_{\beta }^L+bm_{\beta }^L,am_{\beta }^U+bm_{\beta }^U],\\&\qquad [am_{\gamma }^L+bm_{\gamma }^L,am_{\gamma }^U+bm_{\gamma }^U]\rangle \\&\quad =\langle [am_{\alpha }^L,am_{\alpha }^U],[am_{\beta }^L,am_{\beta }^U],\\&\qquad [am_{\gamma }^L,am_{\gamma }^U]\rangle +\langle [bm_{\alpha }^L,bm_{\alpha }^U],\\&\qquad [bm_{\beta }^L,bm_{\beta }^U],[bm_{\gamma }^L,bm_{\gamma }^U]\rangle \\&\quad =a\langle [m_{\alpha }^L,m_{\alpha }^U],[m_{\beta }^L,m_{\beta }^U],\\&\qquad [m_{\gamma }^L,m_{\gamma }^U]\rangle +b\langle [m_{\alpha }^L,m_{\alpha }^U],\\&\qquad [m_{\beta }^L,m_{\beta }^U],[m_{\gamma }^L,m_{\gamma }^U]\rangle \\&\quad =a{\tilde{m}}_{(\alpha ,\beta ,\gamma )}+b{\tilde{m}}_{(\alpha ,\beta ,\gamma )}; \end{aligned}$$
  when $a,b\le 0$, then $a+b\le 0$. The proof of this case of the Lemma is similar to the above case.
2. Let $\lambda > 0$, and then:
  $$\begin{aligned} (\lambda&({\tilde{m}}+{\tilde{n}}))_{(\alpha ,\beta ,\gamma )}\\&\quad =\lambda ({\tilde{m}}+{\tilde{n}})_{(\alpha ,\beta ,\gamma )} \quad [by Proposition\,3.1 (2)]\\&\quad =\lambda ({\tilde{m}}_{(\alpha ,\beta ,\gamma )}\\&\qquad +{\tilde{n}}_{(\alpha ,\beta ,\gamma )}) ~~~~~[by Proposition\, 3.1 (1)]\\&\quad =\lambda \langle [m_{\alpha }^L+n_{\alpha }^L,m_{\alpha }^U+n_{\alpha }^U],\\&\qquad [m_{\beta }^L+n_{\beta }^L,m_{\beta }^U+n_{\beta }^U],\\&\qquad [m_{\gamma }^L+n_{\gamma }^L,m_{\gamma }^U+n_{\gamma }^U]\rangle \\&\quad = \langle [\lambda m_{\alpha }^L+\lambda n_{\alpha }^L,\lambda m_{\alpha }^U+\lambda n_{\alpha }^U],\\&\qquad [\lambda m_{\beta }^L+\lambda n_{\beta }^L,\lambda m_{\beta }^U+\lambda n_{\beta }^U],\\&\qquad [\lambda m_{\gamma }^L+\lambda n_{\gamma }^L,\lambda m_{\gamma }^U+\lambda n_{\gamma }^U]\rangle \\&\quad = \langle [\lambda m_{\alpha }^L,\lambda m_{\alpha }^U],[\lambda m_{\beta }^L,\lambda m_{\beta }^U],[\lambda m_{\gamma }^L,\lambda m_{\gamma }^U]\rangle \\&\qquad + \langle [\lambda n_{\alpha }^L,\lambda n_{\alpha }^U],[\lambda n_{\beta }^L,\lambda n_{\beta }^U],\\&\qquad [\lambda n_{\gamma }^L,\lambda n_{\gamma }^U]\rangle \\&\quad =\lambda {\tilde{m}}_{(\alpha ,\beta ,\gamma )}\\&\qquad +\lambda {\tilde{n}}_{(\alpha ,\beta ,\gamma )}; \end{aligned}$$
  for $\lambda <0$, it is similar to the above case. This completes the proof of this Lemma.

$$\square $$

## Generalized neutrosophic derivative

Moi et al. [44] found some drawback in the Definition 2.6 of neutrosophic derivative. Then, they also define the generalized neutrosophic derivative as follows.

### Definition 4.1

[44] Let $f:I \rightarrow {\mathcal {N}}$ be a neutrosophic-valued function and $x_0 \in I$. Then, the generalized neutrosophic derivative of *f*(*x*) at $x_0$ is denoted by $f'(x_0)$ and defined by:

1. $$f'_{T\alpha }=[min\{f'_{T_1}(x_0;\alpha ),f'_{T_2}(x_0;\alpha )\}, max\{f'_{T_1}(x_0;\alpha ), f'_{T_2}(x_0;\alpha )\}]$$, if $$f'_{T_1}(x_0;\alpha )$$ , $$f'_{T_2}(x_0;\alpha )$$ exists.
2. $$f'_{I\beta }=[min\{f'_{I_1}(x_0;\beta ),f'_{I_2}(x_0;\beta )\}, max\{f'_{I_1}(x_0;\beta ), f'_{I_2}(x_0;\beta )\}]$$, if $$f'_{I_1}(x_0;\beta )$$ , $$f'_{I_2}(x_0;\beta )$$ exists.
3. $$f'_{F\gamma }=[min\{f'_{F_1}(x_0;\gamma ),f'_{F_2}(x_0;\gamma )\}, max\{f'_{F_1}(x_0;\gamma ), f'_{F_2}(x_0;\gamma )\}]$$, if $$f'_{F_1}(x_0;\gamma )$$ , $$f'_{F_2}(x_0;\gamma )$$ exists.

$f'(x)$ is said to be type-1 derivative if $[f'(x_0)]_{(\alpha ,\beta ,\gamma )}=\langle [f'_{T_1}(x_0;\alpha ),f'_{T_2}(x_0;\alpha )],[f'_{I_1}(x_0;\beta ),f'_{I_2}(x_0;\beta )],[f'_{F_1}(x_0;\gamma ),$

$f'_{F_2}(x_0;\gamma )]\rangle$ and type-2 derivative if $[f'(x_0)]_{(\alpha ,\beta ,\gamma )}=\langle [f'_{T_2}(x_0;\alpha ),f'_{T_1}(x_0;\alpha )],[f'_{I_2}(x_0;\beta ),f'_{I_1}(x_0;\beta )],[f'_{F_2}(x_0;\gamma ),$

$$f'_{F_1}(x_0;\gamma )]\rangle $$.

Now, type-1 first-order derivative is denoted by $D_1^1f(x_0)$ and type-2 first-order derivative denoted by $D_2^1f(x_0)$.

In the similar way, we can define another types of derivative of *f*(*x*). Now, $f'(x)$ is said to be:

- type-3 derivative if $[f'(x_0)]_{(\alpha ,\beta ,\gamma )}=\langle [f'_{T_1}(x_0;\alpha ),f'_{T_2}(x_0;\alpha )],[f'_{I_1}(x_0;\beta ),f'_{I_2}(x_0;\beta )],[f'_{F_2}(x_0;\gamma ),f'_{F_1}(x_0;\gamma )]\rangle$
- type-4 derivative if $[f'(x_0)]_{(\alpha ,\beta ,\gamma )}=\langle [f'_{T_1}(x_0;\alpha ),f'_{T_2}(x_0;\alpha )],[f'_{I_2}(x_0;\beta ),f'_{I_1}(x_0;\beta )],[f'_{F_1}(x_0;\gamma ),f'_{F_2}(x_0;\gamma )]\rangle$
- type-5 derivative if $[f'(x_0)]_{(\alpha ,\beta ,\gamma )}=\langle [f'_{T_2}(x_0;\alpha ),f'_{T_1}(x_0;\alpha )],[f'_{I_1}(x_0;\beta ),f'_{I_2}(x_0;\beta )],[f'_{F_1}(x_0;\gamma ),f'_{F_2}(x_0;\gamma )]\rangle$
- type-6 derivative if $[f'(x_0)]_{(\alpha ,\beta ,\gamma )}=\langle [f'_{T_1}(x_0;\alpha ),f'_{T_2}(x_0;\alpha )],[f'_{I_2}(x_0;\beta ),f'_{I_1}(x_0;\beta )],[f'_{F_2}(x_0;\gamma ),f'_{F_1}(x_0;\gamma )]\rangle$
- type-7 derivative if $[f'(x_0)]_{(\alpha ,\beta ,\gamma )}=\langle [f'_{T_2}(x_0;\alpha ),f'_{T_1}(x_0;\alpha )],[f'_{I_1}(x_0;\beta ),f'_{I_2}(x_0;\beta )],[f'_{F_2}(x_0;\gamma ),f'_{F_1}(x_0;\gamma )]\rangle$
- type-8 derivative if $[f'(x_0)]_{(\alpha ,\beta ,\gamma )}=\langle [f'_{T_2}(x_0;\alpha ),f'_{T_1}(x_0;\alpha )],[f'_{I_2}(x_0;\beta ),f'_{I_1}(x_0;\beta )],[f'_{F_1}(x_0;\gamma ),f'_{F_2}(x_0;\gamma )]\rangle$.

However, we will use only type-1 and type-2 derivative of *f*(*x*) in the rest of this article.

### Theorem 4.1

Let ${\mathcal {N}}$ be the set of all neutrosophic number and $f:I\rightarrow {\mathcal {N}}$ be neutrosophic-valued function, where the $(\alpha ,\beta ,\gamma )$-cut of $[f(x)]=\langle [f_{T_1}(x;\alpha ),f_{T_2}(x;\alpha )],[f_{I_1}(x;\beta ),f_{I_2}(x;\beta )],[f_{F_1}(x;\gamma ),f_{F_2}(x;\gamma )]\rangle$, for each $(\alpha ,\beta ,\gamma )$. Then, the Definitions 2.6 and 4.1 of neutrosophic derivative are equivalent.

### Proof

Since $f:I \rightarrow {\mathcal {N}}$ be neutrosophic-valued function. Then, according to the Definition 2.6, $f_{T_1}(x;\alpha )$, $f_{T_2}(x;\alpha )$, $f_{I_1}(x;\beta )$, $f_{I_2}(x;\beta )$, $f_{F_1}(x;\gamma )$, and $f_{F_2}(x;\gamma )$ are differentiable on *I*. If *f* is type-1 differentiable, then $(\alpha ,\beta ,\gamma )$-cut of $[D_1^1f(x)]=\langle [f'_{T_1}(x;\alpha ),f'_{T_2}(x;\alpha )],[f'_{I_1}(x;\beta ),f'_{I_2}(x;\beta )],[f'_{F_1}(x;\gamma ),f'_{F_2}(x;\gamma )]\rangle$, i.e., *f* is generalized neutrosophic differentiable function of type-1. Again, if *f* is type-2 differentiable function, then,$(\alpha ,\beta ,\gamma )$-cut of $[D_2^1f(x)]=\langle [f'_{T_2}(x;\alpha ),f'_{T_1}(x;\alpha )],[f'_{I_2}(x;\beta ),f'_{I_1}(x;\beta )],[f'_{F_2}(x;\gamma ),f'_{F_1}(x_0;\gamma )]\rangle$, i.e., *f* is generalized neutrosophic differentiable function of type-2. Therefore, Definition 2.6 implies Definition 4.1.

Let $f:I\rightarrow {\mathcal {N}}$ is generalized differentiable function of type-1, and then, $f'_{T_1}(x;\alpha )$, $f'_{T_2}(x;\alpha )$, $f'_{I_1}(x;\beta )$, $f'_{I_2}(x;\beta )$, $f'_{F_1}(x;\gamma )$, and $f'_{F_2}(x;\gamma )$ all exist, and the $(\alpha ,\beta ,\gamma )$-cut of $[D_1^1f(x)]=\langle [f'_{T_1}(x;\alpha ),f'_{T_2}(x;\alpha )],[f'_{I_1}(x;\beta ),f'_{I_2}(x;\beta )],$

$$[f'_{F_1}(x;\gamma ),f'_{F_2}(x;\gamma )]\rangle $$.

If $h>0$, then the $(\alpha ,\beta ,\gamma )$-cut of $[f(x+h)-f(x)]$:

$$\begin{aligned}&=\langle [f_{T_1}(x+h;\alpha )-f_{T_1}(x;\alpha ),f_{T_2}(x+h;\alpha )-f_{T_2}(x;\alpha )],\\&\quad [f_{I_1}(x+h;\beta )-f_{I_1}(x;\beta ),f_{I_2}(x+h;\beta )-f_{I_2}(x;\beta )],\\&\quad [f_{F_1}(x+h;\gamma )-f_{F_1}(x;\gamma ),f_{F_2}(x+h;\gamma )-f_{F_2}(x;\gamma )]\rangle \\&\quad [By ~Proposition\, 3.1]. \end{aligned}$$

Multiplying $\dfrac{1}{h}$, then we have from the Definition 4.1 and Proposition 3.1:

$$\begin{aligned}&(\alpha ,\beta ,\gamma )-cut ~of \,\left[ \dfrac{f(x+h)-f(x)}{h}\right] \\&\quad =\left\langle \left[ \dfrac{f_{T_1}(x+h;\alpha )-f_{T_1}(x;\alpha )}{h},\right. \right. \\&\left. \qquad \dfrac{f_{T_2}(x+h;\alpha )-f_{T_2}(x;\alpha )}{h}\right] ,\\&\qquad \left[ \dfrac{f_{I_1}(x+h;\beta )-f_{I_1}(x;\beta )}{h},\right. \\&\qquad \left. \dfrac{f_{I_2}(x+h;\beta ) -f_{I_2}(x;\beta )}{h}\right] ,\\&\qquad \left[ \dfrac{f_{F_1}(x+h;\gamma )-f_{F_1}(x;\gamma )}{h},\right. \\&\left. \left. \qquad \dfrac{f_{F_2}(x+h;\gamma )-f_{F_2}(x;\gamma )}{h}\right] \right\rangle . \end{aligned}$$

Taking limit as $h\rightarrow 0$, we get:

$$\begin{aligned}&(\alpha ,\beta ,\gamma )-cut ~of ~[f'(x)]=\langle [f'_{T_1}(x;\alpha ),f'_{T_2}(x;\alpha )],\\&\quad [f'_{I_1}(x;\beta ),f'_{I_2}(x;\beta )], [f'_{F_1}(x;\gamma ),f'_{F_2}(x;\gamma )]\rangle . \end{aligned}$$

This can be written as:

$$\begin{aligned}&(\alpha ,\beta ,\gamma )-cut ~of ~[D_1^1f(x)]=\langle [f'_{T_1}(x;\alpha ),f'_{T_2}(x;\alpha )],\\&\quad [f'_{I_1}(x;\beta ),f'_{I_2}(x;\beta )], [f'_{F_1}(x;\gamma ),f'_{F_2}(x;\gamma )]\rangle . \end{aligned}$$

Again, *f* is type-2 differentiable. If $h<0$, then:

$$\begin{aligned}&(\alpha ,\beta ,\gamma )-cut ~of \,\left[ f(x+h)-f(x)\right] \\&\quad =\left\langle \left[ f_{T_1}(x+h;\alpha )\right. \right. \\&\left. \qquad -f_{T_1}(x;\alpha ),f_{T_2}(x+h;\alpha )-f_{T_2}(x;\alpha )\right] ,\\&\qquad \left[ f_{I_1}(x+h;\beta )-f_{I_1}(x;\beta ),\right. \\&\left. \qquad f_{I_2}(x+h;\beta )-f_{I_2}(x;\beta )\right] ,\\&\qquad \left[ f_{F_1}(x+h;\gamma )-f_{F_1}(x;\gamma ),\right. \\&\left. \left. \qquad f_{F_2}(x+h;\gamma )-f_{F_2}(x;\gamma )\right] \right\rangle \\&\qquad [By ~Proposition \,3.1]. \end{aligned}$$

Multiplying $\dfrac{1}{-h}$, then we have from the Definition 4.1 and Proposition 3.1:

$$\begin{aligned}&(\alpha ,\beta ,\gamma )-cut ~of ~\left[ \dfrac{f(x+h)-f(x)}{-h}\right] \\&\quad =\left\langle \left[ \dfrac{f_{T_2}(x+h;\alpha )-f_{T_2}(x;\alpha )}{-h},\right. \right. \\&\left. \qquad \dfrac{f_{T_1}(x+h;\alpha )-f_{T_1}(x;\alpha )}{-h}\right] ,\\&\qquad \left[ \dfrac{f_{I_2}(x+h;\beta )-f_{I_2}(x;\beta )}{-h},\right. \\&\left. \qquad \dfrac{f_{I_1}(x+h;\beta )-f_{I_1}(x;\beta )}{-h}\right] ,\\&\qquad \left[ \dfrac{f_{F_2}(x+h;\gamma )-f_{F_2}(x;\gamma )}{-h},\right. \\&\left. \left. \qquad \dfrac{f_{F_1}(x+h;\gamma )-f_{F_1}(x;\gamma )}{-h}\right] \right\rangle . \end{aligned}$$

Taking limit as $h\rightarrow 0$, we get:

$$\begin{aligned}&(\alpha ,\beta ,\gamma )-cut ~of ~[f'(x)]=\langle [f'_{T_2}(x;\alpha ),f'_{T_1}(x;\alpha )],\\&\quad [f'_{I_2}(x;\beta ),f'_{I_1}(x;\beta )], [f'_{F_2}(x;\gamma ),f'_{F_1}(x;\gamma )]\rangle . \end{aligned}$$

This can be written as:

$$\begin{aligned}&(\alpha ,\beta ,\gamma )-cut ~of ~[D_2^1f(x)]=\langle [f'_{T_2}(x;\alpha ),f'_{T_1}(x;\alpha )],\\&\quad [f'_{I_2}(x;\beta ),f'_{I_1}(x;\beta )],[f'_{F_2}(x;\gamma ),f'_{F_1}(x;\gamma )]\rangle . \end{aligned}$$

Therefore, Definition 4.1 implies Definition 2.6.

This completes the proof. $\square$

### Definition 4.2

Let $f':I \rightarrow {\mathcal {N}}$ and $g:I\rightarrow {\mathcal {N}}$ be the neutrosophic-valued function and $g(x)=f'(x)$, $\forall x \in I$, i.e., $(\alpha ,\beta ,\gamma )$-cut of $g(x)=\langle [g_{T_1}(x;\alpha ),g_{T_2}(x;\alpha )],[g_{I_1}(x;\beta ),g_{I_2}(x;\beta )],[g_{F_1}(x;\gamma ),g_{F_2}(x;\gamma )]\rangle$, where $g_{K_1}(x;\delta )=\min \{f'_{K_1}(x;\delta ),f'_{K_2}(x;\delta )\}$, $g_{K_2}(x;\delta )=\max \{f'_{K_1}(x;\delta ),f'_{K_2}(x;\delta )\}$, where $K=T,I$ and *F*, $\delta = \alpha , \beta$ and $\gamma$. Then, the generalized second-order neutrosophic derivative of *f*(*x*) at $x_0\in I$ is denoted and defined by $f''(x_0)=g'(x_0)$:

1. $$g'_{T\alpha }=[min\{g'_{T_1}(x_0;\alpha ),g'_{T_2}(x_0;\alpha )\}, max\{g'_{T_1}(x_0;\alpha ),g'_{T_2}(x_0;\alpha )\}]$$, if $$g'_{T_1}(x_0;\alpha )$$ , $$g'_{T_2}(x_0;\alpha )$$ exists.
2. $$g'_{I\beta }=[min\{g'_{I_1}(x_0;\beta ),g'_{I_2}(x_0;\beta )\}, max\{g'_{I_1}(x_0;\beta ),g'_{I_2}(x_0;\beta )\}]$$, if $$g'_{I_1}(x_0;\beta )$$ , $$g'_{I_2}(x_0;\beta )$$ exists.
3. $$g'_{F\gamma }=[min\{g'_{F_1}(x_0;\gamma ),g'_{F_2}(x_0;\gamma )\}, max\{g'_{F_1}(x_0;\gamma ),g'_{F_2}(x_0;\gamma )\}]$$, if $$g'_{F_1}(x_0;\gamma )$$ , $$g'_{F_2}(x_0;\gamma )$$ exists.

It is said to be type-1 derivative if $(\alpha ,\beta ,\gamma )$-cut of $g'(x_0)=\langle [g'_{T_1}(x_0;\alpha ),g'_{T_2}(x_0;\alpha )],[g'_{I_1}(x_0;\beta ),g'_{I_2}(x_0;\beta )],[g'_{F_1}(x_0;\gamma );$ $g'_{F_2}(x_0;\gamma )]\rangle$ and type-2 derivative if $(\alpha ,\beta ,\gamma )$-cut of $g'(x_0)=\langle [g'_{T_2}(x_0;\alpha ),g'_{T_1}(x_0;\alpha )],[g'_{I_2}(x_0;\beta ),g'_{I_1}(x_0;\beta )],[g'_{F_2}(x_0;\gamma ),$

$$g'_{F_1}(x_0;\gamma )]\rangle $$.

By this similar process, we can define the *nth*-order derivative of a neutrosophic-valued function.

### Definition 4.3

Let $f:I \rightarrow {\mathcal {N}}$ be a neutrosophic-valued function and $n,m=1,2$. Then, *f*(*x*) is said to be (*n*, *m*)-type differentiable at $x_0 \in I$; if $D_n^1f(x_0)$ exists on a neighborhood of $x_0$ as neutrosophic function and it is also m-type differentiable at $x_0$, then second-order neutrosophic derivative of *f*(*x*) at $x_0$ is denoted by $D_{n,m}^2f(x_0)$ for $n,m=1,2$.

### Theorem 4.2

Let $D_1^1f:I\rightarrow {\mathcal {N}}$ or $D_2^1f:I\rightarrow {\mathcal {N}}$ be two neutrosophic functions. Then:

1. If $D_1^1f(x)$ is type-1 differentiable, then $f'_{T_1}(x;\alpha )$, $f'_{T_2}(x;\alpha )$, $f'_{I_1}(x;\beta )$, $f'_{I_2}(x;\beta )$, $f'_{F_1}(x;\gamma )$, and $f'_{F_2}(x;\gamma )$ are all differentiable functions and:
  $$\begin{aligned}&(\alpha ,\beta ,\gamma )-cut ~of~ D_{1,1}^2f(x)=\langle [f''_{T_1}(x;\alpha ),f''_{T_2}(x;\alpha )],\\&\quad [f''_{I_1}(x;\beta ),f''_{I_2}(x;\beta )],[f''_{F_1}(x;\gamma ),f''_{F_2}(x;\gamma )]\rangle . \end{aligned}$$
2. If $D_1^1f(x)$ is type-2 differentiable, then $f'_{T_1}(x;\alpha )$, $f'_{T_2}(x;\alpha )$, $f'_{I_1}(x;\beta )$, $f'_{I_2}(x;\beta )$, $f'_{F_1}(x;\gamma )$, and $f'_{F_2}(x;\gamma )$ are all differentiable functions and:
  $$\begin{aligned}&(\alpha ,\beta ,\gamma )-cut ~of~ D_{1,2}^2f(x)=\langle [f''_{T_2}(x;\alpha ),f''_{T_1}(x;\alpha )],\\&\quad [f''_{I_2}(x;\beta ),f''_{I_1}(x;\beta )],[f''_{F_2}(x;\gamma ),f''_{F_1}(x;\gamma )]\rangle . \end{aligned}$$
3. If $D_2^1f(x)$ is type-1 differentiable, then $f'_{T_1}(x;\alpha )$, $f'_{T_2}(x;\alpha )$, $f'_{I_1}(x;\beta )$, $f'_{I_2}(x;\beta )$, $f'_{F_1}(x;\gamma )$, and $f'_{F_2}(x;\gamma )$ are all differentiable functions and:
  $$\begin{aligned}&(\alpha ,\beta ,\gamma )-cut ~of~ D_{2,1}^2f(x)=\langle [f''_{T_2}(x;\alpha ),f''_{T_1}(x;\alpha )],\\&\quad [f''_{I_2}(x;\beta ),f''_{I_1}(x;\beta )],[f''_{F_2}(x;\gamma ),f''_{F_1}(x;\gamma )]\rangle . \end{aligned}$$
4. If $D_2^1f(x)$ is type-2 differentiable, then $f'_{T_1}(x;\alpha )$, $f'_{T_2}(x;\alpha )$, $f'_{I_1}(x;\beta )$, $f'_{I_2}(x;\beta )$, $f'_{F_1}(x;\gamma )$, and $f'_{F_2}(x;\gamma )$ are all differentiable functions, and:
  $$\begin{aligned}&(\alpha ,\beta ,\gamma )-cut ~of~ D_{2,2}^2f(x)=\langle [f''_{T_1}(x;\alpha ),f''_{T_2}(x;\alpha )],\\&\quad [f''_{I_1}(x;\beta ),f''_{I_2}(x;\beta )],[f''_{F_1}(x;\gamma ),f''_{F_2}(x;\gamma )]\rangle . \end{aligned}$$

### Proof

1. If $h>0$, then $(\alpha ,\beta ,\gamma )$-cut of $[D_1^1f(x+h)-D_1^1f(x)]$ is:
  $$\begin{aligned}&[D_1^1f(x+h)-D_1^1f(x)]\\&\quad =\langle [f'_{T_1}(x+h;\alpha )-f'_{T_1}(x;\alpha ),\\&\qquad f'_{T_2}(x+h;\alpha )-f'_{T_2}(x;\alpha )],\\&\qquad [f'_{I_1}(x+h;\beta )-f'_{I_1}(x;\beta ),\\&\qquad f'_{I_2}(x+h;\beta )-f'_{I_2}(x;\beta )],\\&\qquad [f'_{F_1}(x+h;\gamma )-f'_{F_1}(x;\gamma ),f'_{F_2}(x+h;\gamma )\\&\qquad -f'_{F_2}(x;\gamma )]\rangle \\&\qquad [By ~Proposition \,3.1]. \end{aligned}$$
  Multiplying $\dfrac{1}{h}$, then we have from the Definition 2.6 and Proposition 3.1:
  $$\begin{aligned}&\left[ \dfrac{D_1^1f(x+h)-D_1^1f(x)}{h}\right] _{(\alpha ,\beta ,\gamma )}\\&\quad =\left\langle \left[ \dfrac{f'_{T_1}(x+h;\alpha )-f'_{T_1}(x;\alpha )}{h},\right. \right. \\&\left. \qquad \dfrac{f'_{T_2}(x+h;\alpha )-f'_{T_2}(x;\alpha )}{h}\right] ,\\&\qquad \left[ \dfrac{f'_{I_1}(x+h;\beta )-f'_{I_1}(x;\beta )}{h},\right. \\&\left. \qquad \dfrac{f'_{I_2}(x+h;\beta )-f'_{I_2}(x;\beta )}{h}\right] ,\\&\qquad \left[ \dfrac{f'_{F_1}(x+h;\gamma )-f'_{F_1}(x;\gamma )}{h},\right. \\&\left. \left. \qquad \dfrac{f'_{F_2}(x+h;\gamma )-f'_{F_2}(x;\gamma )}{h}\right] \right. \rangle . \end{aligned}$$
  Taking limit as $h\rightarrow 0$, we get:
  $$\begin{aligned}&(\alpha ,\beta ,\gamma )-cut ~of~ D_1^1f'(x)\\&\quad =\langle [f''_{T_1}(x;\alpha ),f''_{T_2}(x;\alpha )],\\&\qquad [f''_{I_1}(x;\beta ),f''_{I_2}(x;\beta )],\\&\qquad [f''_{F_1}(x;\gamma ),f''_{F_2}(x;\gamma )]\rangle . \end{aligned}$$
  This can be written as:
  $$\begin{aligned}&(\alpha ,\beta ,\gamma )-cut ~of~ D_{1,1}^2f(x)\\&\quad =\langle [f''_{T_1}(x;\alpha ),f''_{T_2}(x;\alpha )],\\&\qquad [f''_{I_1}(x;\beta ),f''_{I_2}(x;\beta )],\\&\qquad [f''_{F_1}(x;\gamma ),f''_{F_2}(x;\gamma )]\rangle . \end{aligned}$$
2. If $$h<0$$, then:
  $$\begin{aligned}&(\alpha ,\beta ,\gamma )-cut ~of~ D_1^1f(x+h)-D_1^1f(x)\\&\quad =\langle [f'_{T_1}(x+h;\alpha )-f'_{T_1}(x;\alpha ),\\&\qquad f'_{T_2}(x+h;\alpha )-f'_{T_2}(x;\alpha )],\\&\qquad [f'_{I_1}(x+h;\beta )-f'_{I_1}(x;\beta ),f'_{I_2}(x+h;\beta )\\&\qquad -f'_{I_2}(x;\beta )],\\&\qquad [f_{F'_1}(x+h;\gamma )-f'_{F_1}(x;\gamma ),\\&\qquad f'_{F_2}(x+h;\gamma )-f'_{F_2}(x;\gamma )]\rangle \\&\qquad [By ~Proposition \,3.1]. \end{aligned}$$
  Multiplying $\dfrac{1}{-h}$, then we have from the Definition 2.6 and Proposition 3.1:
  $$\begin{aligned}&\left[ \dfrac{D_1^1f(x+h)-D_1^1f(x)}{-h}\right] _{(\alpha ,\beta ,\gamma )}\\&\quad =\left\langle \left[ \dfrac{f'_{T_2}(x+h;\alpha )-f'_{T_2}(x;\alpha )}{-h},\right. \right. \\&\left. \qquad \dfrac{f'_{T_1}(x+h;\alpha )-f'_{T_1}(x;\alpha )}{-h}\right] ,\\&\qquad \left[ \dfrac{f'_{I_2}(x+h;\beta )-f'_{I_2}(x;\beta )}{-h},\right. \\&\left. \qquad \dfrac{f'_{I_1}(x+h;\beta )-f'_{I_1}(x;\beta )}{-h}\right] ,\\&\qquad \left[ \dfrac{f'_{F_2}(x+h;\gamma )-f'_{F_2}(x;\gamma )}{-h},\right. \\&\left. \left. \qquad \dfrac{f'_{F_1}(x+h;\gamma )-f'_{F_1}(x;\gamma )}{-h}\right] \right\rangle . \end{aligned}$$
  Taking limit as $h\rightarrow 0$, we get:
  $$\begin{aligned}&(\alpha ,\beta ,\gamma )-cut ~of~ D_2^1f'(x)\\&\quad =\langle [f''_{T_2}(x;\alpha ),f''_{T_1}(x;\alpha )],[f''_{I_2}(x;\beta ),\\&\qquad f''_{I_1}(x;\beta )],[f''_{F_2}(x;\gamma ),f''_{F_1}(x;\gamma )]\rangle . \end{aligned}$$
  This can be written as:
  $$\begin{aligned}&(\alpha ,\beta ,\gamma )-cut ~of~ D_{1,2}^2f(x)\\&\quad =\langle [f''_{T_2}(x;\alpha ),f''_{T_1}(x;\alpha )],[f''_{I_2}(x;\beta ),\\&\qquad f''_{I_1}(x;\beta )],[f''_{F_2}(x;\gamma ),f''_{F_1}(x;\gamma )]\rangle . \end{aligned}$$

The proof the third and fourth part of the theorem is similar to the second and first part, respectively. $\square$

### Theorem 4.3

Let $f:I \rightarrow {\mathcal {N}}$ and $g:I \rightarrow {\mathcal {N}}$ be the neutrosophic differentiable function, such that *f*(*x*) is type-1 differentiable function and *g*(*x*) is type-2 differentiable function on *I*. Then, $(f-g)(x)$ is also differentiable function on *I* and $(f-g)'(x)=f'(x)-g'(x)$. Furthermore:

$$\begin{aligned}&(\alpha ,\beta ,\gamma )-cut ~of~ (f-g)'(x)\\&\quad =\langle [f'_{T_1}(x;\alpha )-g'_{T_2}(x;\alpha ),f'_{T_2}(x;\alpha )-g'_{T_1}(x;\alpha )],\\&\qquad [f'_{I_1}(x;\beta )-g'_{I_2}(x;\beta ),\\&\qquad f'_{I_2}(x;\beta )-g'_{I_1}(x;\beta )],\\&\qquad [f'_{F_1}(x;\gamma )-g'_{F_2}(x;\gamma ),\\&\qquad f'_{F_2}(x;\gamma )-g'_{F_1}(x;\gamma )]\rangle . \end{aligned}$$

### Proof

Since *f* is type-1 differentiable, then we have:

$$\begin{aligned}&\left\langle \left[ \lim _{h \rightarrow 0}\dfrac{f_{T_1}(x+h;\alpha )-f_{T_1}(x;\alpha )}{h},\lim _{h \rightarrow 0}\right. \right. \\&\qquad \left. \dfrac{f_{T_2}(x+h;-f_{T_2}(x;\alpha )}{h}\right] ,\\&\qquad \left[ \lim _{h \rightarrow 0}\dfrac{f_{I_1}(x+h;\beta )-f_{I_1}(x;\beta )}{h},\right. \\&\qquad \left. \lim _{h \rightarrow 0}\dfrac{f_{I_2}(x+h;\beta )-f_{I_2}(x;\beta )}{h}\right] ,\\&\qquad \left[ \lim _{h \rightarrow 0}\dfrac{f_{F_1}(x+h;\gamma )-f_{F_1}(x;\gamma )}{h},\lim _{h \rightarrow 0}\right. \\&\qquad \left. \left. \dfrac{f_{F_2}(x+h;\gamma )-f_{F_2}(x;\gamma )}{h}\right] \right\rangle ; \end{aligned}$$

this limits are exists. Let $i=1,2$, and then, there exists $U_{T_i}(x,h;\alpha )$, $U_{I_i}(x,h;\beta )$, and $U_{F_i}(x,h;\gamma )$, such that:

4.1 $$\begin{aligned} f_{T_i}(x+h;\alpha )&=f_{T_i}(x;\alpha )+U_{T_i}(x,h;\alpha ) \end{aligned}$$

4.2 $$\begin{aligned} f_{I_i}(x+h;\beta )&=f_{I_i}(x;\beta )+U_{T_i}(x,h;\beta ) \end{aligned}$$

4.3 $$\begin{aligned} f_{F_i}(x+h;\gamma )&=f_{F_i}(x;\gamma )+U_{F_i}(x,h;\gamma ). \end{aligned}$$

Since *g* is type-2 differentiable, then there exists $V_{T_i}(x,h;\alpha )$, $V_{I_i}(x,h;\beta )$, and $V_{F_i}(x,h;\gamma )$, such that:

4.4 $$\begin{aligned} g_{T_i}(x;\alpha )&=g_{T_i}(x+h;\alpha )+V_{T_i}(x,h;\alpha ) \end{aligned}$$

4.5 $$\begin{aligned} g_{I_i}(x;\beta )&=g_{I_i}(x+h;\beta )+V_{T_i}(x,h;\beta ) \end{aligned}$$

4.6 $$\begin{aligned} g_{F_i}(x;\gamma )&=g_{F_i}(x+h;\gamma )+V_{F_i}(x,h;\gamma ). \end{aligned}$$

Now, from Eqs. 4.1 and 4.4, we have:

4.7 $$\begin{aligned}&f_{T_i}(x+h;\alpha )+g_{T_i'}(x;\alpha )\nonumber \\&\quad =f_{T_i}(x;\alpha )+g_{T_i'}(x+h;\alpha )\nonumber \\&\qquad +U_{T_i}(x,h;\alpha )+V_{T_i'}(x,h;\alpha ), \end{aligned}$$

where $$i=1,2$$ and $$i'=\{1,2\} {\setminus } i$$.

From Eq. 4.7, we have:

4.8 $$\begin{aligned}&(f_{T_i}(x+h;\alpha )-g_{T_i'}(x+h;\alpha ))-(f_{T_i}(x)-g_{T_i'}(x))\nonumber \\&\quad =U_{T_i}(x,h;\alpha )+V_{T_i'}(x,h;\alpha ). \end{aligned}$$

Since $$\lim _{h \rightarrow 0} \dfrac{U_{T_i}(x,h;\alpha )}{h}\!=\!f'_{T_i}(x;\alpha )$$ and $$ \lim _{h \rightarrow 0} \dfrac{V_{T_i'}(x,h;\alpha )}{h}\!=\!-g'_{T_i'}(x;\alpha )$$.

Now, multiplying Eq. 4.8 by $\dfrac{1}{h}$ and taking limit $h \rightarrow 0$, we have:

$$\begin{aligned} (f_{T_i}-g_{T_i'})'(x;\alpha )=f'_{T_i}(x;\alpha )-g'_{T_i'}(x;\alpha ). \end{aligned}$$

By the similar argument, we have:

$$\begin{aligned} (f_{I_i}-g_{I_i'})'(x;\beta )&=f'_{I_i}(x;\beta )-g'_{I_i'}(x;\beta )\\ (f_{F_i}-g_{F_i'})'(x;\gamma )&=f'_{F_i}(x;\gamma )-g'_{F_i'}(x;\gamma ). \end{aligned}$$

Therefore, $f-g$ is differentiable function and $(f-g)'(x)=f'(x)-g'(x)$, where $i=1,2$ and $i'=\{1,2\} {\setminus } i$.

By similar process, we can show the same result when *f* is type-2 and *g* is type-1 differentiable. $\square$

### Theorem 4.4

Let $f:I \rightarrow {\mathcal {N}}$ and $g:I \rightarrow {\mathcal {N}}$ be two neutrosophic-valued function. Let *f* and *g* are second-order generalized neutrosophic differentiable function on *I*, such that *f* is (1,1)-type and *g* is (2,1)-type differentiable function or *f* is (1,2)-type and *g* is (2,2)-type differentiable function or *f* is (2,1)-type and *g* is (1,1)-type differentiable function or *f* is (2,2)-type and *g* is (1,2)-type differentiable function on *I*. Then, $(f-g)$ is also second-order differentiable function on *I* and:

$$\begin{aligned} (f-g)''(x)=f''(x)-g''(x). \end{aligned}$$

### Proof

For the first case, *f* is (1,1)-type differentiable and *g* is (2,1)-type differentiable. Then, by the above Theorem 4.3, $(f-g)(x)$ is type-1 differentiable and $(f-g)'(x)=f'(x)-g'(x)$. Furthermore:

$$\begin{aligned}&[(f-g)'(x)]_{(\alpha ,\beta ,\gamma )}\\&\quad =\langle [f'_{T_1}(x;\alpha )-g'_{T_2}(x;\alpha ),f'_{T_2}(x;\alpha )-g'_{T_1}(x;\alpha )],\\&\qquad [f'_{I_1}(x;\beta )-g'_{I_2}(x;\beta ),f'_{I_2}(x;\beta )-g'_{I_1}(x;\beta )],\\&\qquad [f'_{F_1}(x;\gamma )-g'_{F_2}(x;\gamma ),\\&\qquad f'_{F_2}(x;\gamma )-g'_{F_1}(x;\gamma )]\rangle . \end{aligned}$$

Then, by Proposition 3.2, $(f-g)(x)$ is (1,1)-type differentiable. Then, we have $(f-g)''(x)=f''(x)-g''(x)$. Furthermore:

$$\begin{aligned}&[(f-g)''(x)]_{(\alpha ,\beta ,\gamma )}\\&\quad =\langle [f''_{T_1}(x;\alpha )-g''_{T_2}(x;\alpha ),\\&\qquad f''_{T_2}(x;\alpha )-g''_{T_1}(x;\alpha )],\\&\qquad [f''_{I_1}(x;\beta )-g''_{I_2}(x;\beta ),\\&\qquad f''_{I_2}(x;\beta )-g''_{I_1}(x;\beta )],\\&\qquad [f''_{F_1}(x;\gamma )-g''_{F_2}(x;\gamma ),\\&\qquad f''_{F_2}(x;\gamma )-g''_{F_1}(x;\gamma )]\rangle . \end{aligned}$$

This completes the proof of the first case of the Theorem. Other cases are similar to the first case. $\square$

### Theorem 4.5

Let $f:I \rightarrow {\mathbb {R}}$ be a real-valued function and $g:I \rightarrow {\mathcal {N}}$ be a neutrosophic-valued function. Then:

1. If $f(x).f'(x)>0$ and *g* is type-1 differentiable, then *f*.*g* is type-1 differentiable and $(f.g)'(x)=f'(x)g(x)+f(x)g'(x)$. Furthermore:
  $$\begin{aligned}&[(f.g)'(x)]_{(\alpha ,\beta ,\gamma )}\\&\quad =\langle [f(x)g'_{T_1}(x;\alpha )+f'(x)g_{T_1}(x;\alpha ),\\&\qquad f(x)g'_{T_2}(x;\alpha )+f'(x)g_{T_2}(x;\alpha )],\\&\qquad [f(x)g'_{I_1}(x;\beta )+f'(x)g_{I_1}(x;\beta ),\\&\qquad f(x)g'_{I_2}(x;\beta )+f'(x)g_{I_2}(x;\beta )],\\&\qquad [f(x)g'_{F_1}(x;\gamma )+f'(x)g_{F_1}(x;\gamma ),\\&\qquad f(x)g'_{F_2}(x;\gamma )+f'(x)g_{F_2}(x;\gamma )]\rangle . \end{aligned}$$
2. If $f(x).f'(x)<0$ and *g* is type-2 differentiable, then *f*.*g* is type-2 differentiable and $(f.g)'(x)=f'(x)g(x)+f(x)g'(x)$. Furthermore:
  $$\begin{aligned}&[(f.g)'(x)]_{(\alpha ,\beta ,\gamma )}\\&\quad =\langle [f(x)g'_{T_2}(x;\alpha )+f'(x)g_{T_2}(x;\alpha ),\\&\qquad f(x)g'_{T_1}(x;\alpha )+f'(x)g_{T_1}(x;\alpha )],\\&\qquad [f(x)g'_{I_2}(x;\beta )+f'(x)g_{I_2}(x;\beta ),\\&\qquad f(x)g'_{I_1}(x;\beta )+f'(x)g_{I_1}(x;\beta )],\\&\qquad [f(x)g'_{F_2}(x;\gamma )+f'(x)g_{F_2}(x;\gamma ),\\&\qquad f(x)g'_{F_1}(x;\gamma )+f'(x)g_{F_1}(x;\gamma )]\rangle . \end{aligned}$$

### Proof

1. There are two subcases. **Subcase 1:** Let $f(x)>0$ and $f'(x)>0$. Since *g* is type-1 differentiable, then:
  $$\begin{aligned}&\left\langle \left[ \lim _{h \rightarrow 0}\dfrac{g_{T_1}(x+h;\alpha )-g_{T_1}(x;\alpha )}{h},\right. \right. \\&\qquad \left. \lim _{h \rightarrow 0}\dfrac{g_{T_2}(x+h;\alpha )-g_{T_2}(x;\alpha )}{h}\right] ,\\&\qquad \left[ \lim _{h \rightarrow 0}\dfrac{g_{I_1}(x+h;\beta )-g_{I_1}(x;\beta )}{h},\right. \\&\qquad \left. \lim _{h \rightarrow 0}\dfrac{g_{I_2}(x+h;\beta )-g_{I_2}(x;\beta )}{h}\right] ,\\&\qquad \left[ \lim _{h \rightarrow 0}\dfrac{g_{F_1}(x+h;\gamma )-g_{F_1}(x;\gamma )}{h},\right. \\&\qquad \left. \left. \lim _{h \rightarrow 0}\dfrac{g_{F_2}(x+h;\gamma )-g_{F_2}(x;\gamma )}{h}\right] \right\rangle ; \end{aligned}$$
  this limits are exists. Let $i=1,2$; then, there exists $U_{T_i}(x,h;\alpha )$, $U_{I_i}(x,h;\beta )$, and $U_{F_i}(x,h;\gamma )$, such that:
  4.9 $$\begin{aligned} g_{T_i}(x+h;\alpha )&=g_{T_i}(x;\alpha )+U_{T_i}(x,h;\alpha ) \end{aligned}$$
  4.10 $$\begin{aligned} g_{I_i}(x+h;\beta )&=g_{I_i}(x;\beta )+U_{T_i}(x,h;\beta ) \end{aligned}$$
  4.11 $$\begin{aligned} g_{F_i}(x+h;\gamma )&=g_{F_i}(x;\gamma )+U_{F_i}(x,h;\gamma ). \end{aligned}$$
  Since $f(x)>0$ and $f'(x)>0$, then we have $f(x+h)=f(x)+V(x,h)$, where $V(x,h)=f(x+h)-f(x)>0$. Now, from Eq. 4.9, we have:
  $$\begin{aligned}&f(x+h).g_{T_i}(x+h;\alpha )=f(x).g_{T_i}(x;\alpha )\\&\quad +f(x)U_{T_i}(x,h;\alpha )+V(x,h)g_{T_i}(x;\alpha )\\&\quad +V(x,h)U_{T_i}(x,h;\alpha ). \end{aligned}$$
  This implies that: $f(x+h).g_{T_i}(x+h;\alpha )-f(x).g_{T_i}(x;\alpha )=f(x)U_{T_i}(x,h;\alpha )+V(x,h)g_{T_i}(x;\alpha )+V(x,h)U_{T_i}(x,h;\alpha )$. Multiplying both side by $\dfrac{1}{h}$ and taking limit as $h \rightarrow 0$. Then, we have:
  $$\begin{aligned} (f.g_{T_i})'(x;\alpha )=f(x)g'_{T_i}(x;\alpha )+f'(x)g_{T_i}(x;\alpha ). \end{aligned}$$
  By similar process, we can find that:
  $$\begin{aligned} (f.g_{I_i})'(x;\beta )&=f(x)g'_{I_i}(x;\beta )+f'(x)g_{I_i}(x;\beta )\\ (f.g_{F_i})'(x;\gamma )&=f(x)g'_{F_i}(x;\gamma )+f'(x)g_{F_i}(x;\gamma ). \end{aligned}$$
  Therefore, $(f.g)'(x)=f'(x)g(x)+f(x)g'(x)$. **Subcase 2:** Let $f(x)<0$ and $f'(x)<0$. Proof of this subcase is similar to the subcase 1.
2. There are two subcases. **Subcase 1:** Let $f(x)<0$ and $f'(x)>0$. Since *g* is type-2 differentiable, then:
  $$\begin{aligned}&\left\langle \left[ \lim _{h \rightarrow 0}\dfrac{g_{T_2}(x;\alpha )-g_{T_1}(x+h;\alpha )}{h},\right. \right. \\&\qquad \left. \lim _{h \rightarrow 0}\dfrac{g_{T_1}(x;\alpha )-g_{T_1}(x+h;\alpha )}{h}\right] ,\\&\qquad \left[ \lim _{h \rightarrow 0}\dfrac{g_{I_2}(x;\beta )-g_{I_2}(x+h;\beta )}{h},\right. \\&\left. \lim _{h \rightarrow 0}\dfrac{g_{I_1}(x;\beta )-g_{I_1}(x+h;\beta )}{h}\right] ,\\&\qquad \left[ \lim _{h \rightarrow 0}\dfrac{g_{F_2}(x;\gamma )-g_{F_2}(x+h;\gamma )}{h},\right. \\&\qquad \left. \left. \lim _{h \rightarrow 0}\dfrac{g_{F_1}(x;\gamma )-g_{F_1}(x+h;\gamma )}{h}\right] \right\rangle ; \end{aligned}$$
  this limits are exists. Let $i=1,2$, and then, there exists $V_{T_i}(x,h;\alpha )$, $V_{I_i}(x,h;\beta )$, and $V_{F_i}(x,h;\gamma )$, such that:
  4.12 $$\begin{aligned} g_{T_i}(x;\alpha )&=g_{T_i}(x+h;\alpha )+V_{T_i}(x,h;\alpha ) \end{aligned}$$
  4.13 $$\begin{aligned} g_{I_i}(x;\beta )&=g_{I_i}(x+h;\beta )+V_{T_i}(x,h;\beta ) \end{aligned}$$
  4.14 $$\begin{aligned} g_{F_i}(x;\gamma )&=g_{F_i}(x+h;\gamma )+V_{F_i}(x,h;\gamma ). \end{aligned}$$
  Since $f(x)<0$ and $f'(x)>0$, then we have $f(x)=f(x+h)+V(x,h)$, where $V(x,h)=f(x)-f(x+h)<0$. Now, from Eq. 4.12, we have: $f(x).g_{T_i}(x;\alpha )=f(x+h).g_{T_i}(x+h;\alpha )+f(x+h)V_{T_i}(x,h;\alpha )\!+\!V(x,h)g_{T_i}(x\!+\!h;\alpha )\!+\!V(x,h)V_{T_i}(x,h;\alpha )$. This implies that: $f(x).g_{T_i}(x;\alpha )-f(x+h).g_{T_i}(x+h;\alpha )=f(x+h)V_{T_i}(x,h;\alpha )+V(x,h)g_{T_i}(x+h;\alpha )+V(x,h)V_{T_i}(x,h;\alpha )$ Multiplying both side by $-\dfrac{1}{h}$ and taking limit as $h \rightarrow 0$. Then, we have:
  $$\begin{aligned} (f.g_{T_i})'(x;\alpha )=f(x)g'_{T_i}(x;\alpha )+f'(x)g_{T_i}(x;\alpha ). \end{aligned}$$
  By similar process, we can find that:
  $$\begin{aligned} (f.g_{I_i})'(x;\beta )&=f(x)g'_{I_i}(x;\beta )+f'(x)g_{I_i}(x;\beta )\\ (f.g_{F_i})'(x;\gamma )&=f(x)g'_{F_i}(x;\gamma )+f'(x)g_{F_i}(x;\gamma ), \end{aligned}$$
  where $i=1,2$. Therefore, $(f.g)'(x)=f'(x)g(x)+f(x)g'(x)$. **Subcase 2:** Let $f(x)>0$ and $f'(x)<0$. Proof of this subcase is similar to the subcase 1.

This completes the proof. $\square$

## Neutrosophic boundary-value problem

Let us consider the second-order neutrosophic boundary-value problem as follows:

5.1 $$\begin{aligned} \left. \begin{aligned} y''(x)=f(x,y(x),y'(x))\\ y=a ~at ~x=0~i.e~y(0)=a\\ y=b ~at ~x=1~i.e~ y(1)=b \end{aligned} \right\} , \end{aligned}$$

where *a* and *b* are neutrosophic number, and $f:[0,1]\times {\mathcal {N}}\times {\mathcal {N}} \rightarrow {\mathcal {N}}$ be a neutrosophic function.

### Definition 5.1

Let $y:[0,1]\rightarrow {\mathcal {N}}$ be a neutrosophic function and $n,m \in \{1,2\}$. Then, *y* is said to be (*n*, *m*)-solution of Eq. 5.1 on [0, 1] if $D_n^1y$, $D_{n,m}^2y$ exists on [0, 1] and $D_{n,m}^2y(x)=f(x,y(x),D_n^1y(x))$, $y(0)=a$, $y(1)=b$.

To find the solution of the neutrosophic boundary-value problem 5.1, we can translate Eq. 5.1 to the system of boundary-value problems.

Therefore, there are four types of possible system of boundary-value problem.

**(1,1) System :**

$$\begin{aligned}&y''_{T_i}(x;\alpha )=f_{T_i}(x,y_{T_1}(x;\alpha ),y_{T_{2}}(x;\alpha ),\\&\quad y'_{T_1}(x;\alpha ),y'_{T_{2}}(x;\alpha ))\\&y''_{I_i}(x;\beta )=f_{I_i}(x,y_{I_1}(x;\beta ),y_{I_{2}}(x;\beta ),\\&\quad y'_{I_1}(x;\beta ),y'_{I_{2}}(x;\beta ))\\&y''_{F_i}(x;\gamma )=f_{F_i}(x,y_{F_1}(x;\alpha ),\\&\quad y_{F_{2}}(x;\alpha ),y'_{F_1}(x;\alpha ),y'_{F_{2}}(x;\gamma )) \end{aligned}$$

with the boundary conditions

$$y_{T_i}(0;\alpha )=a_{T_i}^{\alpha }$$    $$y_{I_i}(0;\beta )=a_{I_i}^{\beta }$$    $$y_{F_i}(0;\gamma )=a_{F_i}^{\gamma }$$ $$y_{T_i}(1;\alpha )=b_{T_i}^{\alpha }$$    $$y_{I_i}(1;\beta )=a_{I_i}^{\beta }$$    $$y_{F_i}(1;\gamma )=a_{F_i}^{\gamma }$$,

where $$i=1,2$$ and $$i'=\{1,2\} {\setminus } i$$.

Here, $D_1^1y$, $D_{1,1}^2y$ exists and $(\alpha ,\beta ,\gamma )$-cut of $D_1^1y(x)=\langle [y'_{T_1}(x;\alpha ),y'_{T_2}(x;\alpha )],[y'_{I_1}(x;\beta ),y'_{I_2}(x;\beta )],[y'_{F_1}(x;\gamma ),y'_{F_2}(x;\gamma )]\rangle$ and $(\alpha ,\beta ,\gamma )$-cut of $D_{1,1}^2y(x)=\langle [y''_{T_1}(x;\alpha ),y''_{T_2}(x;\alpha )],[y''_{I_1}(x;\beta ),y''_{I_2}(x;\beta )],[y''_{F_1}(x;\gamma ),y''_{F_2}(x;\gamma )]\rangle$.

Since there is no derivative involve in the boundary conditions. Therefore, for all remaining system, the boundary conditions will be same as above. Only the equation will be change.

**(1,2) System :**

$$\begin{aligned}&y''_{T_{i'}}(x;\alpha )=f_{T_i}(x,y_{T_1}(x;\alpha ),\\&\quad y_{T_{2}}(x;\alpha ),y'_{T_1}(x;\alpha ),y'_{T_{2}}(x;\alpha ))\\&\quad y''_{I_{i'}}(x;\beta )=f_{I_i}(x,y_{I_1}(x;\beta ),\\&\quad y_{I_{2}}(x;\beta ),y'_{I_1}(x;\beta ),y'_{I_{2}}(x;\beta ))\\&\quad y''_{F_{i'}}(x;\gamma )=f_{F_i}(x,y_{F_1}(x;\gamma ),\\&\quad y_{F_{2}}(x;\gamma ),y'_{F_1}(x;\gamma ),y'_{F_{2}}(x;\gamma )), \end{aligned}$$

where $$i=1,2$$ and $$i'=\{1,2\} {\setminus } i$$.

Here, $D_1^1y$, $D_{1,2}^2y$ exists, and $(\alpha ,\beta ,\gamma )$-cut of $D_1^1y(x)=\langle [y'_{T_1}(x;\alpha ),y'_{T_2}(x;\alpha )],[y'_{I_1}(x;\beta ),y'_{I_2}(x;\beta )],[y'_{F_1}(x;\gamma ),y'_{F_2}(x;\gamma )]\rangle$ and $(\alpha ,\beta ,\gamma )$-cut of $D_{1,2}^2y(x)=\langle [y''_{T_2}(x;\alpha ),y''_{T_1}(x;\alpha )],[y''_{I_2}(x;\beta ),y''_{I_1}(x;\beta )],[y''_{F_2}(x;\gamma ),y''_{F_1}(x;\gamma )]\rangle$.

**(2,1) System :**

$$\begin{aligned}&y''_{T_{i'}}(x;\alpha )=f_{T_i}(x,y_{T_1}(x;\alpha ),\\&\quad y_{T_{2}}(x;\alpha ),y'_{T_1}(x;\alpha ),y'_{T_{2}}(x;\alpha ))\\&\quad y''_{I_{i'}}(x;\beta )=f_{I_i}(x,y_{I_1}(x;\beta ),\\&\quad y_{I_{2}}(x;\beta ),y'_{I_1}(x;\beta ),y'_{I_{2}}(x;\beta ))\\&\quad y''_{F_{i'}}(x;\gamma )=f_{F_i}(x,y_{F_1}(x;\gamma ),\\&\quad y_{F_{2}}(x;\gamma ),y'_{F_1}(x;\gamma ),y'_{F_{2}}(x;\gamma )), \end{aligned}$$

where $$i=1,2$$ and $$i'=\{1,2\} {\setminus } i$$.

Here, $$D_2^1y$$, $$D_{2,1}^2y$$ exists and $$(\alpha ,\beta ,\gamma )$$-cut of $$D_2^1y(x)=\langle [y'_{T_2}(x;\alpha ),y'_{T_1}(x;\alpha )],[y'_{I_2}(x;\beta ),y'_{I_1}(x;\beta )],$$$$[y'_{F_2}(x;\gamma ),y'_{F_1}(x;\gamma )]\rangle $$ and $$(\alpha ,\beta ,\gamma )$$-cut of $$D_{2,1}^2y(x)=\langle [y''_{T_2}(x;\alpha ),y''_{T_1}(x;\alpha )],[y''_{I_2}(x;\beta ),y''_{I_1}(x;\beta )],[y''_{F_2}(x;\gamma ),y''_{F_1}(x;\gamma )]\rangle $$.

**(2,2) System :**

$$\begin{aligned}&y''_{T_i}(x;\alpha )=f_{T_i}(x,y_{T_1}(x;\alpha ),\\&\quad y_{T_{2}}(x;\alpha ),y'_{T_1}(x;\alpha ),y'_{T_{2}}(x;\alpha ))\\&\quad y''_{I_i}(x;\beta )=f_{I_i}(x,y_{I_1}(x;\beta ),\\&\quad y_{I_{2}}(x;\beta ),y'_{I_1}(x;\beta ),y'_{I_{2}}(x;\beta ))\\&\quad y''_{F_i}(x;\gamma )=f_{F_i}(x,y_{F_1}(x;\alpha ),\\&\quad y_{F_{2}}(x;\alpha ),y'_{F_1}(x;\alpha ),y'_{F_{2}}(x;\gamma )), \end{aligned}$$

where $$i=1,2$$ and $$i'=\{1,2\} {\setminus } i$$.

Here, $D_2^1y$, $D_{2,2}^2y$ exists and $(\alpha ,\beta ,\gamma )$-cut of $D_2^1y(x)=\langle [y'_{T_2}(x;\alpha ),y'_{T_1}(x;\alpha )],[y'_{I_2}(x;\beta ),y'_{I_1}(x;\beta )],[y'_{F_2}(x;\gamma ),y'_{F_1}(x;\gamma )]\rangle$ and $(\alpha ,\beta ,\gamma )$-cut of $D_{2,2}^2y(x)=\langle [y''_{T_1}(x;\alpha ),y''_{T_2}(x;\alpha )],[y''_{I_1}(x;\beta ),y''_{I_2}(x;\beta )],[y''_{F_1}(x;\gamma ),y''_{F_2}(x;\gamma )]\rangle$.

Let *y* be the (*n*, *m*)-solution of the boundary-value problem 5.1 at $x_0 \in [0,1]$. Therefore, $[y(x_0)]_{(\alpha ,\beta ,\gamma )}=\langle [y_{T_1}(x_0;\alpha ),y_{T_2}(x_0;\alpha )].$

$[y_{I_1}(x_0;\beta ),y_{I_2}(x_0;\beta )],[y_{F_1}(x_0;\gamma ),y_{F_2}(x_0;\gamma )]\rangle$. Then, $D_n^1y$ and $D_{n,m}^2y$ exists at $x_0$ and it satisfies Eq. 5.1. Using this facts, we are giving a table to show that when the solutions are exists at $x_0$.

**Table:** 

System	*y*	$y'$	$y''$
(1,1)	$y_{K_1}\le y_{K_2}$	$y'_{K_1}\le y'_{K_2}$	$y''_{K_1}\le y''_{K_2}$
(1,2)	$y_{K_1}\le y_{K_2}$	$y'_{K_1}\le y'_{K_2}$	$y''_{K_2}\le y''_{K_1}$
(2,1)	$y_{K_1}\le y_{K_2}$	$y'_{K_2}\le y'_{K_1}$	$y''_{K_2}\le y''_{K_1}$
(2,2)	$y_{K_1}\le y_{K_2}$	$y'_{K_2}\le y'_{K_1}$	$y''_{K_1}\le y''_{K_2}$

where $$K=T,I$$ and *F*.

## Examples

In this section, we shall discuss some test problems and their numerical results. The Tables are calculated using Wolfram Mathematica 9.0 and the figures have been drawn using MATLAB R2018a. The Matlab code for the figures has been given below.

**Matlab code :**

1. x=k; %Choose k, where $$x\in (0,1)$$.
2. $a_1=$linespace(a,b,c); %Choose the linespace of membership value, where (a,b) be the interval where       membership value lies and c be the line spacing.
3. $y=f_1(x;a_1);$ %Put the function.
4. plot($y,a_1$,’color’) %For plotting the function y.
5. hold on.
6. $z=f_2(x;a_1);$ %Put the another function.
7. plot($z,a_1$,’color’) %For plotting the function z.
8. hold off.
9. xlabel(’x’); %For x-axis.
10. ylabel(’y’); %For y-axis.

### Example 1

Let us consider second-order neutrosophic boundary-value problem as follows:

$$\begin{aligned}&y''(x)=2{\tilde{a}}, ~~y(0)=\dfrac{1}{8}{\tilde{a}},\quad \\&\quad y(1)=\dfrac{3}{8}{\tilde{a}}, \end{aligned}$$

where ${\tilde{a}}=\langle (-1,0,1); 0.6,0.4,0.2\rangle$ is a single-valued triangular neutrosophic number. Then, $(\alpha ,\beta ,\gamma )$-level set of ${\tilde{a}}$ is ${\tilde{a}}_{(\alpha ,\beta ,\gamma )}=\langle [\dfrac{5\alpha -3}{3},\dfrac{3-5\alpha }{3}],[\dfrac{2-5\beta }{3},\dfrac{5\beta -2}{3}],[\dfrac{1-5\gamma }{4},\dfrac{5\gamma -1}{4}]\rangle$, where $\alpha \in [0,0.6]$, $\beta \in [0.4,1]$ and $\gamma \in [0.2,1]$. Now, we try to find out the solution of the boundary-value problem for (1, 1) system, (1, 2) system, (2, 1) system, and (2, 2) system.

**(1,1) System**

If *y* is a (1, 1) solution for the boundary-value problem, then:

$$\begin{aligned}&[y'(x)]_{(\alpha ,\beta ,\gamma )}\\&\quad =\langle [y'_{T_1}(x;\alpha ),y'_{T_2}(x;\alpha )],\\&\qquad [y'_{I_1}(x;\beta ),y'_{I_2}(x;\beta )],\\&\qquad [y'_{F_1}(x;\gamma ),y'_{F_2}(x;\gamma )]\rangle \\&\qquad [y''(x)]_{(\alpha ,\beta ,\gamma )}\\&\quad =\langle [y''_{T_1}(x;\alpha ),y''_{T_2}(x;\alpha )],\\&\qquad [y''_{I_1}(x;\beta ),y''_{I_2}(x;\beta )],\\&\qquad [y''_{F_1}(x;\gamma ),y''_{F_2}(x;\gamma )]\rangle \end{aligned}$$

Then, the boundary-value problem can be written in the form as follows:

$$\begin{aligned}&[y''(x)]_{(\alpha ,\beta ,\gamma )}\\&\quad =\left\langle \left[ \dfrac{10\alpha -6}{3},\dfrac{6-10\alpha }{3}\right] ,\right. \\&\qquad \left[ \dfrac{4-10\beta }{3},\dfrac{10\beta -4}{3}\right] ,\\&\left. \qquad \left[ \dfrac{1-5\gamma }{2},\dfrac{5\gamma -1}{2}\right] \right\rangle \\&[y(0)]_{(\alpha ,\beta ,\gamma )}=\left\langle \left[ \dfrac{5\alpha -3}{24},\right. \right. \\&\left. \qquad \dfrac{3-5\alpha }{24}\right] ,\left[ \dfrac{2-5\beta }{24},\right. \\&\left. \qquad \dfrac{5\beta -2}{24}\right] ,\left[ \dfrac{1-5\gamma }{32},\right. \\&\left. \left. \qquad \dfrac{5\gamma -1}{32}\right] \right\rangle \\&[y(1)]_{(\alpha ,\beta ,\gamma )}=\left\langle \left[ \dfrac{5\alpha -3}{8},\right. \right. \\&\left. \qquad \dfrac{3-5\alpha }{8}\right] ,\\&\qquad \left[ \dfrac{2-5\beta }{8},\dfrac{5\beta -2}{8}\right] ,\\&\left. \qquad \left[ \dfrac{3-15\gamma }{32},\dfrac{15\gamma -3}{32}\right] \right\rangle . \end{aligned}$$

Therefore, the solution of the (1, 1) system is:

6.1 $$\begin{aligned} \left. \begin{aligned} y_{T_1}(x;\alpha )=\dfrac{5\alpha -3}{24}(8x^2-6x+1), ~~~y_{T_2}(x;\alpha )=\dfrac{3-5\alpha }{24}(8x^2-6x+1) \\ y_{I_1}(x;\beta )=\dfrac{2-5\beta }{24}(8x^2-6x+1), ~~~y_{I_2}(x;\beta )=\dfrac{5\beta -2}{24}(8x^2-6x+1) \\ y_{F_1}(x;\gamma )=\dfrac{1-5\gamma }{32}(8x^2-6x+1), ~~~y_{F_2}(x;\gamma )=\dfrac{5\gamma -1}{32}(8x^2-6x+1). \end{aligned} \right\} \end{aligned}$$

Therefore, the (1,1)-solution of the boundary-value problem can written as follows:

$$\begin{aligned}&[y(x)]_{(\alpha ,\beta ,\gamma )}\\&\quad =\left\langle \left[ \dfrac{5\alpha -3}{24}(8x^2-6x+1),\right. \right. \\&\left. \qquad \dfrac{3-5\alpha }{24}(8x^2-6x+1)\right] ,\\&\qquad \left[ \dfrac{2-5\beta }{24}(8x^2-6x+1),\right. \\&\qquad \left. \dfrac{5\beta -2}{24}(8x^2-6x+1)\right] ,\\&\qquad \left[ \dfrac{1-5\gamma }{32}(8x^2-6x+1),\right. \\&\left. \left. \qquad \dfrac{5\gamma -1}{32}(8x^2-6x+1)\right] \right\rangle . \end{aligned}$$

The solution of the problem is shown in Fig. 2. Then, the solution gives a neutrosophic number if $8x^2-6x+1 \ge 0$. Hence, its represents a neutrosophic number for $x\ge \dfrac{1}{2}$ and $x\le \dfrac{1}{4}$.

Then, the type-1 derivative of the solution is:

$$\begin{aligned}&[D_1^1y(x)]_{(\alpha ,\beta ,\gamma )}\\&\quad =\left\langle \left[ \dfrac{5\alpha -3}{24}(16x-6),\right. \right. \\&\qquad \left. \dfrac{3-5\alpha }{24}(16x-6)\right] ,\\&\qquad \left[ \dfrac{2-5\beta }{24}(16x-6),\right. \\&\left. \qquad \dfrac{5\beta -2}{24}(16x-6)\right] ,\\&\qquad \left[ \dfrac{1-5\gamma }{32}(16x-6),\right. \\&\left. \left. \qquad \dfrac{5\gamma -1}{32}(16x-6)\right] \right\rangle . \end{aligned}$$

It gives a neutrosophic number for $x\ge \dfrac{1}{2}$. Then, it is again type-1 differentiable and:

$$\begin{aligned}&[D_{1,1}^2y(x)]_{(\alpha ,\beta ,\gamma )}\\&\quad =\left\langle \left[ \dfrac{10\alpha -6}{3},\right. \right. \\&\left. \qquad \dfrac{6-10\alpha }{3}\right] ,\left[ \dfrac{4-10\beta }{3},\right. \\&\left. \qquad \dfrac{10\beta -4}{3}\right] ,\left[ \dfrac{1-5\gamma }{2},\right. \\&\qquad \left. \left. \dfrac{5\gamma -1}{2}\right] \right\rangle . \end{aligned}$$

However, for $x\le \dfrac{1}{4}$, it is not a type-1 differentiable. Therefore, the *y* is (1, 1) differentiable for $x\ge \dfrac{1}{2}$. Then, $D_1^1y$ and $D_{1,1}^2y$ exist for $x\in (\dfrac{1}{2},1)$. Therefore, *y* gives a (1, 1) solution of the neutrosophic boundary-value problem on $(\dfrac{1}{2},1)$.

**Fig. 1:**
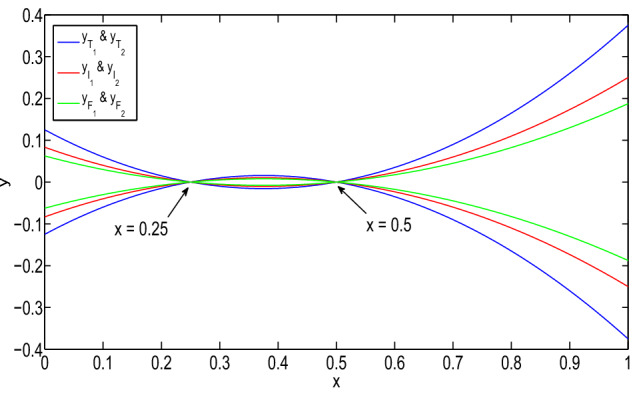
(1,1)-solution and (2,2)-solution of Example 1 for $\alpha =0$, $\beta =0.8$, and $\gamma =0.6$

**Fig. 2:**
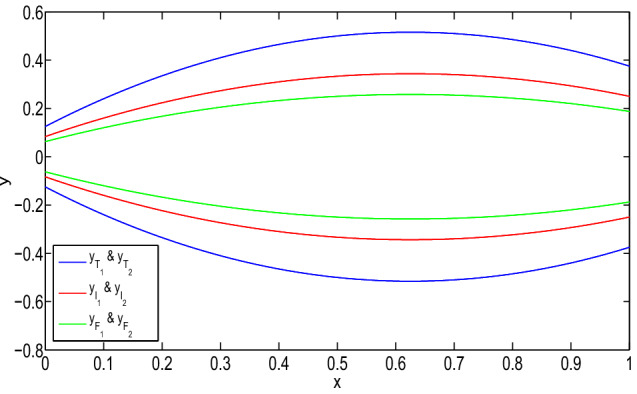
(1,2)-solution and (2,1)-solution of Example 1 for $\alpha =0$, $\beta =0.8$, and $\gamma =0.6$

**(2,2) System**

The solution of the boundary-value problem for (2,2) system is:

$$\begin{aligned}&[y(x)]_{(\alpha ,\beta ,\gamma )}\\&\quad =\left\langle \left[ \dfrac{5\alpha -3}{24}(8x^2-6x+1),\right. \right. \\&\qquad \left. \dfrac{3-5\alpha }{24}(8x^2-6x+1)\right] ,\\&\qquad \left[ \dfrac{2-5\beta }{24}(8x^2-6x+1),\right. \\&\left. \qquad \dfrac{5\beta -2}{24}(8x^2-6x+1)\right] ,\\&\qquad \left[ \dfrac{1-5\gamma }{32}(8x^2-6x+1),\right. \\&\left. \left. \qquad \dfrac{5\gamma -1}{32}(8x^2-6x+1)\right] \right\rangle . \end{aligned}$$

The solution of the problem is shown in Fig. 2. Then, the solution gives a neutrosophic number if $8x^2-6x+1 \ge 0$. Hence, its represents a neutrosophic number for $x\ge \dfrac{1}{2}$ and $x\le \dfrac{1}{4}$

For $x\le \dfrac{1}{4}$, *y* is type-2 differentiable. Then, type-2 derivative of *y* is:

$$\begin{aligned}&[D_2^1y(x)]_{(\alpha ,\beta ,\gamma )}\\&\quad =\left\langle \left[ \dfrac{3-5\alpha }{24}(16x-6),\right. \right. \\&\left. \qquad \dfrac{5\alpha -3}{24}(16x-6)\right] ,\left[ \dfrac{5\beta -2}{24}(16x-6),\right. \\&\left. \qquad \dfrac{2-5\beta }{24}(16x-6)\right] ,\\&\qquad \left[ \dfrac{5\gamma -1}{32}(16x-6),\right. \\&\left. \left. \qquad \dfrac{1-5\gamma }{32}(16x-6)\right] \right\rangle . \end{aligned}$$

Since it gives a neutrosophic number for $x\le \dfrac{1}{4}$. Therefore, *y* is type-2 differentiable. Then, we have:

$$\begin{aligned}&[D_{2,2}^2y(x)]_{(\alpha ,\beta ,\gamma )}\\&\quad =\left\langle \left[ \dfrac{10\alpha -6}{3},\dfrac{6-10\alpha }{3}\right] ,\right. \\&\qquad \left[ \dfrac{4-10\beta }{3},\dfrac{10\beta -4}{3}\right] ,\\&\qquad \left. \left[ \dfrac{1-5\gamma }{2},\dfrac{5\gamma -1}{2}\right] \right\rangle . \end{aligned}$$

Therefore, *y* gives a (2, 2) solution on $(0,\dfrac{1}{4})$.

**(1,2) System**

The solution of the boundary-value problem for (1,2) system is:

$$\begin{aligned}&[y(x)]_{(\alpha ,\beta ,\gamma )}\\&\quad =\left\langle \left[ -\dfrac{5\alpha -3}{24}(8x^2-10x-1),\right. \right. \\&\left. \qquad -\dfrac{3-5\alpha }{24}(8x^2-10x-1)\right] ,\\&\qquad \left[ -\dfrac{2-5\beta }{24}(8x^2-10x-1),\right. \\&\left. \qquad -\dfrac{5\beta -2}{24}(8x^2-10x-1)\right] ,\\&\qquad \left[ -\dfrac{1-5\gamma }{32}(8x^2-10x-1),\right. \\&\qquad \left. \left. -\dfrac{5\gamma -1}{32}(8x^2-10x-1)\right] \right\rangle . \end{aligned}$$

The solution for this system is shown in Fig. 2, since its gives a neutrosophic number for $x\in (0,1)$. Then, type-1 derivative of *y* is:

$$\begin{aligned}&[D_1^1y(x)]_{(\alpha ,\beta ,\gamma )}\\&\quad =\left\langle \left[ -\dfrac{5\alpha -3}{24}(16x-10),\right. \right. \\&\left. \qquad -\dfrac{3-5\alpha }{24}(16x-10)\right] ,\\&\qquad \left[ -\dfrac{2-5\beta }{24}(16x-10),-\dfrac{5\beta -2}{24}(16x-10)\right] ,\\&\qquad \left[ -\dfrac{1-5\gamma }{32}(16x-10),\right. \\&\qquad \left. \left. -\dfrac{5\gamma -1}{32}(16x-10)\right] \right\rangle . \end{aligned}$$

Since it gives a neutrosophic number if $x\le \dfrac{5}{8}$. Then, it is also type-2 differentiable. Therefore, we have:

$$\begin{aligned}&[D_{1,2}^2y(x)]_{(\alpha ,\beta ,\gamma )}\\&\quad =\left\langle \left[ \dfrac{10\alpha -6}{3},\dfrac{6-10\alpha }{3}\right] ,\right. \\&\qquad \left[ \dfrac{4-10\beta }{3},\dfrac{10\beta -4}{3}\right] ,\\&\qquad \left. \left[ \dfrac{1-5\gamma }{2},\dfrac{5\gamma -1}{2}\right] \right\rangle . \end{aligned}$$

Therefore, *y* gives a (1, 2) solution on $(0,\dfrac{5}{8})$.

**(2,1) System :**

The solution of the boundary-value problem for (2,1) system is:

$$\begin{aligned}&[y(x)]_{(\alpha ,\beta ,\gamma )}\\&\quad =\left\langle \left[ -\dfrac{5\alpha -3}{24}(8x^2-10x-1),\right. \right. \\&\left. \qquad -\dfrac{3-5\alpha }{24}(8x^2-10x-1)\right] ,\\&\qquad \left[ -\dfrac{2-5\beta }{24}(8x^2-10x-1),\right. \\&\qquad \left. -\dfrac{5\beta -2}{24}(8x^2-10x-1)\right] ,\\&\qquad \left[ -\dfrac{1-5\gamma }{32}(8x^2-10x-1),\right. \\&\qquad \left. \left. -\dfrac{5\gamma -1}{32}(8x^2-10x-1)\right] \right\rangle . \end{aligned}$$

The solution for this system is shown in Fig. 2. Since its gives a neutrosophic number for $x\in (0,1)$. Then, type-2 derivative of *y* is:

$$\begin{aligned}&[D_2^1y(x)]_{(\alpha ,\beta ,\gamma )}\\&\quad =\left\langle \left[ -\dfrac{3-5\alpha }{24}(16x-10),\right. \right. \\&\left. \qquad -\dfrac{5\alpha -3}{24}(16x-10)\right] ,\\&\qquad \left[ -\dfrac{5\beta -2}{24}(16x-10),\right. \\&\left. \qquad -\dfrac{2-5\beta }{24}(16x-10)\right] ,\\&\qquad \left[ -\dfrac{5\gamma -1}{32}(16x-10),\right. \\&\left. \left. \qquad -\dfrac{1-5\gamma }{32}(16x-10)\right] \right\rangle . \end{aligned}$$

Since it gives a neutrosophic number if $x\ge \dfrac{5}{8}$. Then, it is also type-1 differentiable. Therefore, we have:

$$\begin{aligned}&[D_{2,1}^2y(x)]_{(\alpha ,\beta ,\gamma )}\\&\quad =\left\langle \left[ \dfrac{10\alpha -6}{3},\dfrac{6-10\alpha }{3}\right] ,\right. \\&\qquad \left[ \dfrac{4-10\beta }{3},\dfrac{10\beta -4}{3}\right] ,\\&\qquad \left. \left[ \dfrac{1-5\gamma }{2},\dfrac{5\gamma -1}{2}\right] \right\rangle . \end{aligned}$$

Therefore, *y* gives a (1, 2) solution on $(0,\dfrac{5}{8})$

Therefore, the boundary-value problem gives (1, 1)-solution on $(\dfrac{1}{2},1)$, (2, 2)-solution on $(0,\dfrac{1}{4})$, (1, 2)-solution on $(0,\dfrac{5}{8})$, and (2, 1)-solution on $(\dfrac{5}{8},1)$.

**Table 1:** (1,1)-solutions for the different values of $\alpha ,\beta$, and $\gamma$ at $x=3/4$ for Example 1

$\alpha$	$y_{T_1}(x;\alpha )$	$y_{T_2}(x;\alpha )$	$\beta$	$y_{I_1}(x;\beta )$	$y_{I_2}(x;\beta )$	$\gamma$	$y_{F_1}(x;\gamma )$	$y_{F_2}(x;\gamma )$
0	$-$0.125	0.125	0.4	0	0	0.2	0	0
0.2	$-$ 0.0833333	0.0833333	0.6	$-$ 0.0416667	0.0416667	0.4	$-$ 0.03125	0.03125
0.4	$-$ 0.0416667	0.041667	0.8	$-$ 0.0833333	0.0833333	0.6	$-$ 0.0625	0.0625
0.6	0	0	1	$-$ 0.125	0.125	1	$-$ 0.125	0.125

**Table 2:** (1,2)-solutions for the different values of $\alpha ,\beta$, and $\gamma$ at $x=5/16$ for Example 1

$\alpha$	$y_{T_1}(x;\alpha )$	$y_{T_2}(x;\alpha )$	$\beta$	$y_{I_1}(x;\beta )$	$y_{I_2}(x;\beta )$	$\gamma$	$y_{F_1}(x;\gamma )$	$y_{F_2}(x;\gamma )$
0	$-$ 0.417969	0.417969	0.4	0	0	0.2	0	0
0.2	$-$ 0.278646	0.278646	0.6	$-$ 0.139323	0.139323	0.4	$-$ 0.104492	0.104492
0.4	$-$ 0.139323	0.139323	0.8	$-$ 0.278646	0.278646	0.6	$-$ 0.208984	0.208984
0.6	0	0	1	$-$ 4.17969	4.17969	1	$-$ 0.417969	0.417969

**Fig. 3:**
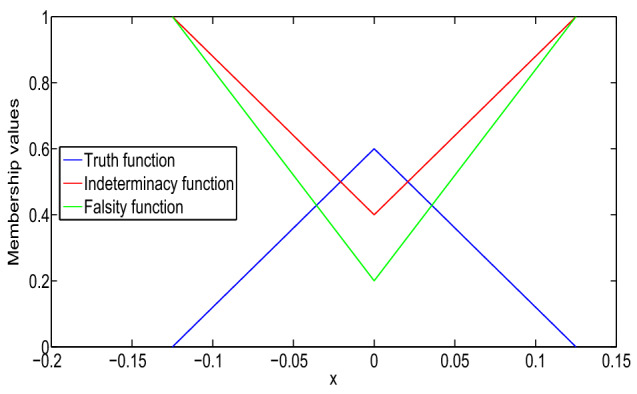
Truth, indeterminacy, and falsity-membership function for (1,1)-solution at $x=3/4$

**Fig. 4:**
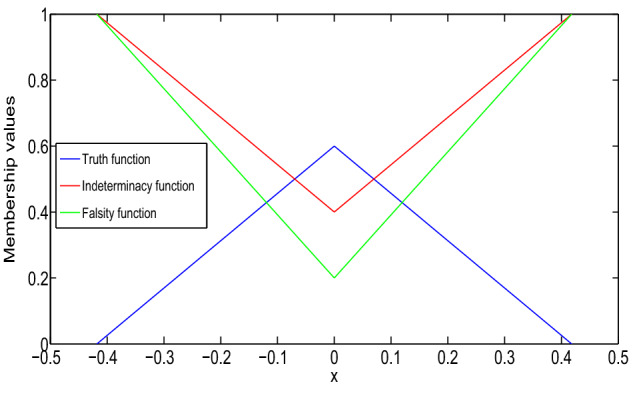
Truth, indeterminacy, and falsity-membership function for (1,2)-solution at $x=5/16$

In Fig. 1, it has been seen that (1,1)-solution and (2,2)-solution of Example 1 exist only for $x\in (1/2,1)$ and $x\in (0,1/4)$, respectively, where $\alpha =0, \beta =0.8$ and $\gamma =0.6$. Also, from Fig. 2, it has been seen that (1,2)-solution and (2,1)-solution of Example 1 exist for $x\in (0,1)$, where $\alpha =0, \beta =0.8$ and $\gamma =0.6$. Therefore, even though the crisp solution exist, but some times, there are some values of x for which the neutrosophic solution does not exist. In Tables 1 and 2, when the value of $\alpha$ increases, then the solution of left branch for truth membership function increases and the solution of right branch for truth membership function decreases. Again, when $\beta$ and $\gamma$ increases, the solution of left branch for indeterminacy and falsity-membership function decreases and the solution of right branch increases. At $\alpha =0.6$, right and left branch of truth membership function gives the same solution. Similarly, at $\beta =0.4$ and $\gamma =0.2$, right and left branch of indeterminacy and falsity-membership function gives the same solution, respectively. This shows that the solution in Tables 1 and 2 for (1,1) and (1,2) system, respectively, gives a neutrosophic number, and from Figs. 3 and 4, it also has been seen that the solution gives a triangular neutrosophic number.

### Example 2

Let us consider second-order neutrosophic boundary-value problem as follows:

$$\begin{aligned} y''(x)={\tilde{a}}, ~~y(0)={\tilde{0}},~~ y(1)={\tilde{b}}, \end{aligned}$$

where ${\tilde{a}}=\langle (0,1,2); 0.6,0.4,0.2\rangle$ and ${\tilde{b}}=\langle (-1,0,1); 0.6,0.4,0.2\rangle$ are single-valued triangular neutrosophic number. Then: ${\tilde{a}}_{(\alpha ,\beta ,\gamma )}=\langle [\dfrac{5\alpha }{3},\dfrac{6-5\alpha }{3}],[\dfrac{5(1-\beta )}{3},\dfrac{5\beta +1}{3}],[\dfrac{5(1-\gamma )}{4},\dfrac{5\gamma +3}{4}]\rangle$ and

${\tilde{b}}_{(\alpha ,\beta ,\gamma )}=\langle [\dfrac{5\alpha -3}{3},\dfrac{3-5\alpha }{3}],[\dfrac{2-5\beta }{3},\dfrac{5\beta -2}{3}],[\dfrac{1-5\gamma }{4},\dfrac{5\gamma -1}{4}]\rangle$ where $\alpha \in [0,0.6]$, $\beta \in [0.4,1]$, and $\gamma \in [0.2,1]$. Now, we try to find out the solution of the boundary-value problem for (1, 1) system, (1, 2) system, (2, 1) system, and (2, 2) system.

**(1,1) System**

The solution of the boundary-value problem for (1,1)-system is:

$$\begin{aligned}&[y(x)]_{(\alpha ,\beta ,\gamma )}\\&\quad =\left\langle \left[ \dfrac{5\alpha }{6}x^2+\dfrac{5\alpha -6}{6}x,\right. \right. \\&\left. \qquad \dfrac{6-5\alpha }{6}x^2-\dfrac{5\alpha }{6}x\right] ,\\&\qquad \left[ \dfrac{5(1-\beta )}{6}x^2-\dfrac{5\beta +1}{6}x,\right. \\&\left. \qquad \dfrac{5\beta +1}{6}x^2+\dfrac{5(\beta -1)}{6}x\right] ,\\&\qquad \left[ \dfrac{5(1-\gamma )}{8}x^2-\dfrac{3+5\gamma }{8}x,\right. \\&\left. \left. \qquad \dfrac{5\gamma +3}{8}x^2+\dfrac{5(\gamma -1)}{8}x\right] \right\rangle . \end{aligned}$$

**Fig. 5:**
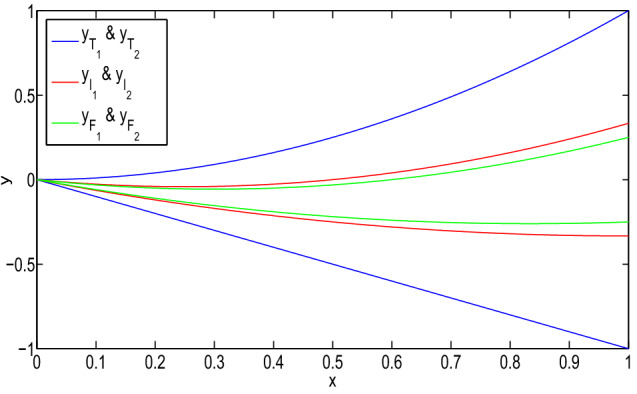
(1,1)-solution of Example 2 for $\alpha =0$, $\beta =0.6$, and $\gamma =0.4$

The solution of the problem is shown in Fig. 5. Then, type-1 derivative of the solution is:

$$\begin{aligned}&[D_1^1y(x)]_{(\alpha ,\beta ,\gamma )}\\&\quad =\left\langle \left[ \dfrac{5\alpha }{3}x+\dfrac{5\alpha -6}{6},\right. \right. \\&\left. \qquad \dfrac{6-5\alpha }{3}x-\dfrac{5\alpha }{6}\right] ,\\&\qquad \left[ \dfrac{5(1-\beta )}{3}x-\dfrac{5\beta +1}{6},\right. \\&\left. \qquad \dfrac{5\beta +1}{3}x+\dfrac{5(\beta -1)}{6}\right] ,\\&\qquad \left[ \dfrac{5(1-\gamma )}{4}x-\dfrac{3+5\gamma }{8},\right. \\&\left. \left. \qquad \dfrac{5\gamma +3}{4}x+\dfrac{5(\gamma -1)}{8}\right] \right\rangle . \end{aligned}$$

It is again type-1 differentiable and:

$$\begin{aligned}&[D_{1,1}^2y(x)]_{(\alpha ,\beta ,\gamma )}\\&\quad =\left\langle \left[ \dfrac{5\alpha }{3},\dfrac{6-5\alpha }{3}\right] ,\right. \\&\qquad \left[ \dfrac{5(1-\beta )}{3},\dfrac{5\beta +1}{3}\right] ,\\&\qquad \left. \left[ \dfrac{5(1-\gamma )}{4},\dfrac{5\gamma +3}{4}\right] \right\rangle . \end{aligned}$$

**(1,2) System**

(1,2)-solution of the boundary-value problem is:

$$\begin{aligned}&[y(x)]_{(\alpha ,\beta ,\gamma )}\\&\quad =\left\langle \left[ \dfrac{6-5\alpha }{6}x^2+\dfrac{5\alpha -4}{2}x,\right. \right. \\&\left. \qquad \dfrac{5\alpha }{6}x^2+\dfrac{2-5\alpha }{2}x\right] ,\\&\qquad \left[ \dfrac{5\beta +1}{6}x^2+\dfrac{1-5\beta }{2}x,\right. \\&\left. \qquad \dfrac{5(1-\beta )}{6}x^2+\dfrac{5\beta -3}{2}x\right] ,\\&\qquad \left[ \dfrac{5\gamma -3}{8}x^2-\dfrac{1+15\gamma }{8}x,\right. \\&\qquad \left. \left. \dfrac{5(1-\gamma )}{8}x^2+\dfrac{15\gamma -7}{8}x\right] \right\rangle . \end{aligned}$$

**Fig. 6:**
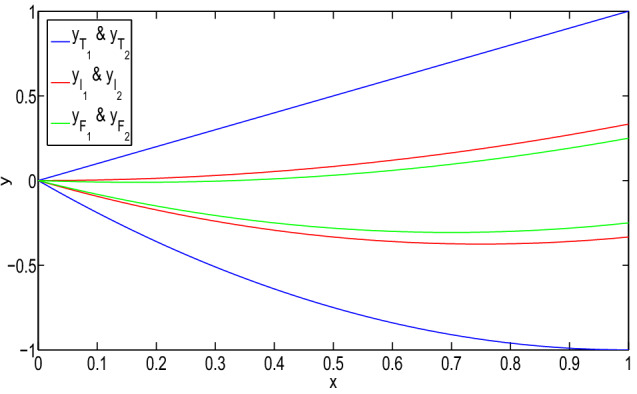
(1,2)-solution of Example 2 for $\alpha =0$, $\beta =0.6$, and $\gamma =0.4$

The solutions for (1,2) system are shown in Fig. 6.

**(2,1) System**

(2,1)-solution for this boundary-value problem does not exist. Because, if (2,1)-solution exists, then the solution can be written in the form as follows:

$$\begin{aligned}&[y(x)]_{(\alpha ,\beta ,\gamma )}\\&\quad =\left\langle \left[ \dfrac{6-5\alpha }{6}x^2+\dfrac{5\alpha -4}{2}x,\right. \right. \\&\left. \qquad \dfrac{5\alpha }{6}x^2+\dfrac{2-5\alpha }{2}x\right] ,\\&\qquad \left[ \dfrac{5\beta +1}{6}x^2+\dfrac{1-5\beta }{2}x,\right. \\&\left. \qquad \dfrac{5(1-\beta )}{6}x^2+\dfrac{5\beta -3}{2}x\right] ,\\&\qquad \left[ \dfrac{5\gamma -3}{8}x^2-\dfrac{1+15\gamma }{8}x,\right. \\&\left. \left. \qquad \dfrac{5(1-\gamma )}{8}x^2+\dfrac{15\gamma -7}{8}x\right] \right\rangle . \end{aligned}$$

Since it is type-2 differentiable. Then:

$$\begin{aligned}&[y'(x)]_{(\alpha ,\beta ,\gamma )}\\&\quad =\left\langle \left[ \dfrac{5\alpha }{3}x+\dfrac{2-5\alpha }{2},\right. \right. \\&\qquad \left. \dfrac{6-5\alpha }{3}x+\dfrac{5\alpha -4}{2}\right] ,\\&\qquad \left[ \dfrac{5(1-\beta )}{3}x+\dfrac{5\beta -3}{2},\right. \\&\left. \qquad \dfrac{5\beta +1}{3}x+\dfrac{1-5\beta }{2}\right] ,\\&\qquad \left[ \dfrac{5(1-\gamma )}{4}x+\dfrac{15\gamma -7}{8},\right. \\&\left. \left. \qquad \dfrac{5\gamma -3}{4}x-\dfrac{1+15\gamma }{8}\right] \right\rangle . \end{aligned}$$

Then, for the Truth membership function, this forms an interval if $\dfrac{5\alpha }{3}x+\dfrac{2-5\alpha }{2}<\dfrac{6-5\alpha }{3}x+\dfrac{5\alpha -4}{2}$. This implies that $x>\dfrac{3}{2}$. Therefore, $x \notin [0,1]$. Therefore, (2,1)-solution does not exists.

**(2,2) System**

By similar argument, we can show that (2,2)-solution does not exist.

**Table 3:** (1,1)-solutions for the different values of $\alpha ,\beta$, and $\gamma$ at $x=1/2$ for Example 2

$\alpha$	$y_{T_1}(x;\alpha )$	$y_{T_2}(x;\alpha )$	$\beta$	$y_{I_1}(x;\beta )$	$y_{I_2}(x;\beta )$	$\gamma$	$y_{F_1}(x;\gamma )$	$y_{F_2}(x;\gamma )$
0	$-$ 0.5	0.25	0.4	$-$ 0.125	$-$ 0.125	0.2	$-$ 0.125	$-$ 0.125
0.2	$-$ 0.375	0.125	0.6	$-$ 0.25	0	0.4	$-$ 0.21875	$-$ 0.03125
0.4	$-$ 0.25	0	0.8	$-$ 0.375	0.125	0.6	$-$ 0.3125	0.0625
0.6	$-$ 0.125	$-$ 0.125	1	$-$ 0.5	0.25	1	$-$ 0.5	0.25

**Fig. 7:**
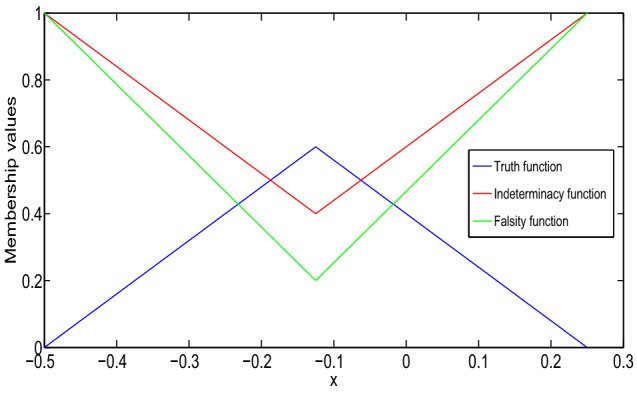
Truth, indeterminacy, and falsity-membership function for (1,1)-solution at $x=1/2$

In Figs. 5 and 6, it has been seen that (1,1)-solution and (1,2)-solution of Example 2 exist for $x\in (0,1)$, where $\alpha =0, \beta =0.6$ and $\gamma =0.4$. From Table 3, we have found similar type of result for Example 2 which was found for Example 1 from Tables 1 and 2. Therefore, from Table 3, it follows that the (1,1)-solution for Example 2 gives a neutrosophic number, and from Fig. 7, it has been seen that the solution gives a triangular neutrosophic number. By similar argument, we can show that the (1,2)-solution for Example 2 gives a neutrosophic number.

### Example 3

Let us consider second-order neutrosophic boundary-value problem as follows:

$$\begin{aligned} y''(x)+2y'(x)+y(x)={\tilde{a}}e^x, ~~y(0)={\tilde{0}},~~ y(1)={\tilde{b}}, \end{aligned}$$

where ${\tilde{a}}=\langle (0,1,2); 0.6,0.4,0.2\rangle$ and ${\tilde{b}}=\langle (-1,0,1); 0.6,0.4,0.2\rangle$ are single-valued triangular neutrosophic number. Then, ${\tilde{a}}_{(\alpha ,\beta ,\gamma )}=\langle [\dfrac{5\alpha }{3},\dfrac{6-5\alpha }{3}],[\dfrac{5(1-\beta )}{3},\dfrac{5\beta +1}{3}],[\dfrac{5(1-\gamma )}{4},\dfrac{5\gamma +3}{4}]\rangle$ and

${\tilde{b}}_{(\alpha ,\beta ,\gamma )}=\langle [\dfrac{5\alpha -3}{3},\dfrac{3-5\alpha }{3}],[\dfrac{2-5\beta }{3},\dfrac{5\beta -2}{3}],[\dfrac{1-5\gamma }{4},\dfrac{5\gamma -1}{4}]\rangle$ where $\alpha \in [0,0.6]$, $\beta \in [0.4,1]$ and $\gamma \in [0.2,1]$. Now, we try to find out the solution of the boundary-value problem for (1, 1) system, (1, 2) system, (2, 1) system, and (2, 2) system.

**(1,1) System**

The solution of the boundary-value problem for (1,1)-system is:

$$\begin{aligned}&[y(x)]_{(\alpha ,\beta ,\gamma )}=\left\langle \left[ y_{T_1}(x;\alpha ),\right. \right. \\&\left. \qquad y_{T_2}(x;\alpha )\right] ,\left[ y_{I_1}(x;\beta ),y_{I_2}(x;\beta )\right] ,\\&\left. \qquad \left[ y_{F_1}(x;\gamma ),y_{F_2}(x;\gamma )\right] \right\rangle , \end{aligned}$$

where:

$$\begin{aligned}&y_{T_1}(x;\alpha )=\left( -\dfrac{5\alpha }{12}\right. \\&\left. \qquad +\dfrac{(5\alpha -3)e}{3}x+\dfrac{5\alpha (1-e^2)}{12}x\right) e^{-x}\\&\qquad +\dfrac{5\alpha e^x}{12}\\&y_{T_2}(x;\alpha )=\left( -\dfrac{6-5\alpha }{12}+\dfrac{(3-5\alpha )e}{3}x\right. \\&\left. \qquad +\dfrac{(6-5\alpha )(1-e^2)}{12}x\right) e^{-x}\\&\qquad +\dfrac{(6-5\alpha ) e^x}{12}\\&y_{I_1}(x;\beta )=\dfrac{5(1-\beta )}{12}(e^x-e^{-x})\\&\qquad +\left( \dfrac{(2-5\beta )e}{3}+\dfrac{5(1-\beta )}{12}(1-e^2)\right) xe^{-x}\\&y_{I_2}(x;\beta )=\dfrac{5\beta +1}{12}(e^x-e^{-x})\\&\qquad +\left( \dfrac{(5\beta -2)e}{3}\right. \\&\left. \qquad +\dfrac{5\beta +1}{12}(1-e^2)\right) xe^{-x}\\&y_{F_1}(x;\gamma )=\dfrac{5(1-\gamma )}{16}(e^x-e^{-x})\\&\qquad +\left( \dfrac{(1-5\gamma )e}{4}\right. \\&\left. \qquad +\dfrac{5(1-\gamma )}{16}(1-e^2)\right) xe^{-x}\\&y_{F_2}(x;\gamma )=\dfrac{5\gamma +3}{16}(e^x-e^{-x})\\&\qquad +\left( \dfrac{(5\gamma -1)e}{4}+\dfrac{5\gamma +3}{16}(1-e^2)\right) xe^{-x}. \end{aligned}$$

**Fig. 8:**
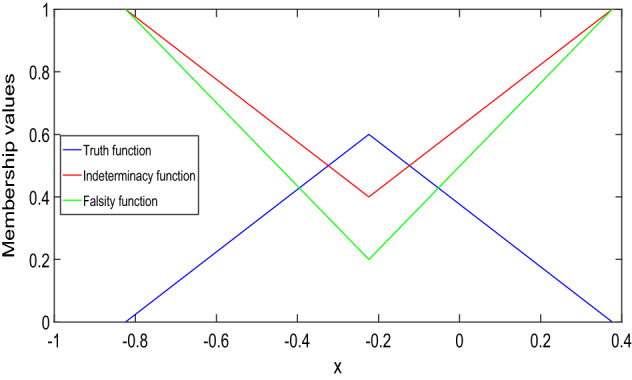
Truth, indeterminacy, and falsity-membership function for (1,1)-solution at $x=1/2$

We see that *y*, $D_1^{1}y$, and $D_{1,1}^2y$ give a valid neutrosophic number for $x\in (0,1)$. Therefore, y is (1,1)-differentiable and it gives a (1,1) solution, which are shown in Fig. 8 and Table 4.

**Table 4:** (1,1)-solutions for the different values of $\alpha ,\beta$, and $\gamma$ at $x=1/2$ for Example 2

$\alpha$	$y_{T_1}(x;\alpha )$	$y_{T_2}(x;\alpha )$	$\beta$	$y_{I_1}(x;\beta )$	$y_{I_2}(x;\beta )$	$\gamma$	$y_{F_1}(x;\gamma )$	$y_{F_2}(x;\gamma )$
0	$-$ 0.824	0.377	0.4	$-$ 0.224	$-$ 0.224	0.2	$-$ 0.224	$-$ 0.224
0.2	$-$ 0.624	0.176	0.6	$-$ 0.424	$-$ 0.024	0.4	$-$ 0.374	$-$ 0.074
0.4	$-$ 0.424	$-$ 0.024	0.8	$-$ 0.624	0.176	0.6	$-$ 0.524	$-$ 0.076
0.6	$-$ 0.224	$-$ 0.224	1	$-$ 0.824	0.377	1	$-$ 0.824	0.377

**(1,2) system**

The solution for (1,2) system is:

$$\begin{aligned}&y_{T_1}(x;\alpha )=\dfrac{1}{12}((10\alpha -3)e^{2-\sqrt{2}}+3e^2-12e\\&\qquad +20e\alpha -10e^2\alpha )xe^{-x}\\&\qquad +\dfrac{3-10\alpha }{12}e^{(1-\sqrt{2})x}+\dfrac{10\alpha -3}{12}e^x\\&y_{T_2}(x;\alpha )=\dfrac{1}{12}((9-10\alpha )e^{2-\sqrt{2}}\\&\qquad -9e^2+12e-20e\alpha +10e^2\alpha )xe^{-x}\\&\qquad +\dfrac{10\alpha -9}{12}e^{(1-\sqrt{2})x}+\dfrac{9-10\alpha }{12}e^x\\&y_{I_1}(x;\beta )=\dfrac{1}{12}(-(10\beta +7)e^{2-\sqrt{2}}\\&\qquad +8e-20e\beta +7e^2+10e^2\beta )xe^{-x}\\&\qquad +\dfrac{1}{12}(7+10\beta )e^{(1-\sqrt{2})x}-\dfrac{20\beta +14}{24}e^x\\&y_{I_2}(x;\beta )=\dfrac{1}{12}((10\beta -1)e^{2-\sqrt{2}}\\&\qquad -8e+20e\beta +e^2-10e^2\beta )xe^{-x}\\&\qquad +\dfrac{1}{12}(1-10\beta )e^{(1-\sqrt{2})x}-\dfrac{2-20\beta }{24}e^x\\&y_{F_1}(x;\gamma )=\dfrac{1}{8}((3-5\gamma )e^{2-\sqrt{2}}\\&\qquad +2e-10e\gamma -3e^2+5e^2\gamma )xe^{-x}\\&\qquad +\dfrac{1}{8}(5\gamma -3)e^{(1-\sqrt{2})x}-\dfrac{5\gamma -3}{8}e^x\\&y_{F_2}(x;\gamma )=\dfrac{1}{8}((1+5\gamma )e^{2-\sqrt{2}}\\&\qquad -2e+10e\gamma -e^2-5e^2\gamma )xe^{-x}\\&\qquad +\dfrac{1}{8}(5\gamma +1)e^{(1-\sqrt{2})x}+\dfrac{5\gamma +1}{8}e^x. \end{aligned}$$

Here, $D_1^1y$ exist, but $D_{1,2}^2y$ does not exist. Therefore, (1,2)-solution does not exist.

By this similar process, we can show that for (2,1) system and (2,2) system, $D_2^1y$, $D_{2,1}^2y$, $D_2^1y$ and $D_{2,2}^2y$ do not exist for $x\in (0,1)$. Therefore (2,1)-solution and (2,2)-solution does not exist.

From Fig. 8 and Table 4, it have been seen that (1,1)-solution of Example 3 exists for $x\in (0,1)$, where $\alpha =0, \beta =0.6$ and $\gamma =0.4$. Also, from Table 4, it has been seen that the (1,1)-solution gives a neutrosophic number, and from Fig.  8, it has been shown that the solution gives a triangular neutrosophic number when the parameters are taken as triangular neutrosophic number.

## Conclusion

In this article, mainly, we have focused on the development of neutrosophic differential equation. Some properties of neutrosophic number have been presented here. In Definition 4.1, we have defined different types of neutrosophic derivative. From Theorem 4.1, it has been seen that the neutrosophic derivative [26] and generalized neutrosophic derivative [44] are equivalent. In Definition 4.2, we have defined different types of (*n*, *m*) differentiability of neutrosophic-valued function, where $n,m\in \{1,2\}$. From Theorems 4.3 and 4.4, it has been seen that the subtraction of two first-order or second-order neutrosophic differentiable functions is also differentiable. In Theorem 4.5, it has been seen that the multiplication of two neutrosophic differentiable function is also differentiable.

Here, we have considered different types of derivatives in the form of different (*n*, *m*) system, where $n,m\in \{1,2\}$. In the first example, the solution of neutrosophic boundary-value problem exists for all (1, 1), (1, 2), (2, 1), and (2, 2) systems, but, from Fig. 1, it has been seen that the (1, 1) and (2, 2) solutions for first example exist only for $x\ge 1/2$ and $x\le 1/4$, respectively. In the second example, it has been seen that the solution of neutrosophic boundary-value problem exists for (1, 1) and (1, 2) systems, but the solution does not exist at all for (2, 1) and (2, 2) systems. Also, in the third example, it has been seen that the (1,1) solution exists, but the solution does not exist for (1,2), (2,1), and (2,2) systems. Therefore, it can be concluded that sometimes (n,m)-solutions for neutrosophic boundary-value problem may exist for all $x\in (0,1)$, where $n,m \in \{1,2\}$; sometimes, it may exist only for some points, and sometimes, it may not exist at all. In Example 1, 2, and 3, from Figs. 3, 4, 7, and 8, it can be concluded that, if we consider all the parameters of a neutrosophic boundary-value problem in the form of triangular neutrosophic number and if the solution exists for any (n,m) systems, then the solutions also give a triangular neutrosophic number for each value of *x*.

## References

[1] Chang Sheldon SL, Zadeh Lofti A (1996) On fuzzy mapping and control. In Fuzzy sets, fuzzy logic, and fuzzy systems: selected papers by Lotfi A Zadeh, pages 180–184. World Scientific

[2] Zadeh LA (1975). The concept of a linguistic variable and its application to approximate reasoning—i. Inf Sci.

[3] Atanassov Krassimir T (1999) Intuitionistic fuzzy sets. In: Intuitionistic fuzzy sets, Springer, New York, pp 1–137

[4] Atanassov KT (1986). Intuitionistic fuzzy sets. Fuzzy Sets Syst.

[5] Smarandache Florentin (2003) Proceedings of the First International Conference on Neutrosophy, Neutrosophic Logic, Neutrosophic Set, Neutrosophic Porbability and Statistics: Www.Gallup.Unm.Edu/ Smarandache/NeutrosophicProceedings.Pdf. Infinite Study

[6] Smarandache F (1999) A unifying field in logics: Neutrosophic logic

[7] Smarandache F (2005). Neutrosophic set-a generalization of the intuitionistic fuzzy set. Int J Pure Appl Math.

[8] Agboola AAA, Akinleye SA (2014). Neutrosophic vector spaces. Neutrosophic Sets Syst.

[9] Salama AA, Alblowi SA (2012). Neutrosophic set and neutrosophic topological spaces. IOSR J Math.

[10] Shabir M, Ali M, Naz M, Smarandache F (2013). Soft neutrosophic group. Neutrosophic Sets Syst.

[11] Agboola AAA, Adeleke EO, Akinleye SA (2012). Neutrosophic rings ii. Int J Math Combin.

[12] Sumathi IR, Priya VM (2018) A new perspective on neutrosophic differential equation. Infinite Study

[13] Sumathi IR, Sweety CAC (2019). New approach on differential equation via trapezoidal neutrosophic number. Complex Intell Syst.

[14] Topal S, Taş F (2018) Bézier surface modeling for neutrosophic data problems. Infinite Study

[15] Broumi S, Arindam D, Bakali A, Talea M, Smarandache F, Son LH, Koley D (2017) Uniform single valued neutrosophic graphs. Infinite study

[16] Broumi S, Son LH, Bakali A, Talea M, Smarandache F, Selvachandran G (2017). Computing operational matrices in neutrosophic environments: a matlab toolbox. Neutrosophic Sets Syst.

[17] Broumi S, Nagarajan D, Bakali A, Talea M, Smarandache F, Lathamaheswari M (2019). The shortest path problem in interval valued trapezoidal and triangular neutrosophic environment. Complex Intell Syst.

[18] Broumi S, Dey A, Talea M, Bakali A, Smarandache F, Nagarajan D, Lathamaheswari M, Kumar R (2019). Shortest path problem using bellman algorithm under neutrosophic environment. Complex Intell Syst.

[19] Broumi S, Talea M, Bakali A, Smarandache F, Nagarajan D, Lathamaheswari M, Parimala M (2019). Shortest path problem in fuzzy, intuitionistic fuzzy and neutrosophic environment: an overview. Complex Intell Syst.

[20] Saranya S, Vigneshwaran M (2019). Net framework to deal with neutrosophic-closed sets in neutrosophic topological spaces. Neutrosophic Sets Syst.

[21] Gulistan M, Khan S (2019) Extentions of neutrosophic cubic sets via complex fuzzy sets with application. Complex Intell Syst pp 1–12

[22] Du S, Ye J, Yong R, Zhang F (2020) Some aggregation operators of neutrosophic z-numbers and their multicriteria decision making method. Complex Intell Syst, pp 1–10 10.1007/s40747-020-00204-w PMC7603794 34777954

[23] Aslam M (2019). A new attribute sampling plan using neutrosophic statistical interval method. Complex & Intelligent Systems.

[24] Edalatpanah SA (2020). A direct model for triangular neutrosophic linear programming. Int J Neutrosophic Sci.

[25] Salama AA, Fazaa M, Yahya M, Kazim M (2020). A suggested diagnostic system of corona virus based on the neutrosophic systems and deep learning. IJNS.

[26] Smarandache F (2015) Neutrosophic precalculus and neutrosophic calculus: neutrosophic applications. Infinite Study,

[27] Son NTK, Dong NP, Long HV, Khastan A (2020). Linear quadratic regulator problem governed by granular neutrosophic fractional differential equations. ISA Trans.

[28] Smarandache F (2013) Introduction to neutrosophic measure, neutrosophic integral, and neutrosophic probability. Infinite Study

[29] Kaleva O (1987). Fuzzy differential equations. Fuzzy Sets Syst.

[30] Seikkala S (1987). On the fuzzy initial value problem. Fuzzy Sets Syst.

[31] Bede B, Gal SG (2005). Generalizations of the differentiability of fuzzy-number-valued functions with applications to fuzzy differential equations.. Fuzzy Sets Syst.

[32] Lakshmikantham V, Murty KN, Turner J (2001). Two-point boundary value problems associated with non-linear fuzzy differential equations. Math Inequal Appl.

[33] Regan DO, Lakshmikantham V, Nieto JJ (2003). Initial and boundary value problems for fuzzy differential equations. Nonlinear Anal Theory Methods Appl.

[34] Bede B (2006). A note on “two-point boundary value problems associated with non-linear fuzzy differential equations”. Fuzzy Sets Syst.

[35] Ma M, Friedman M, Kandel A (1999). Numerical solutions of fuzzy differential equations. Fuzzy Sets Syst.

[36] Abbasbandy S, Viranloo TA (2002). Numerical solution of fuzzy differential equation. Math Comput Appl.

[37] Bede B (2008). Note on “numerical solutions of fuzzy differential equations by predictor-corrector method”. Inf Sci.

[38] Khastan A, Nieto JJ (2010). A boundary value problem for second order fuzzy differential equations. Nonlinear Anal Theory Methods Appl.

[39] Tapaswini S, Chakraverty S (2019). Numerical solution of fuzzy differential equations using orthogonal polynomials. Int J Comput Sci Math.

[40] Balakrishnan S, Manigandan P Numerical solutions of fuzzy differential equations by fifth order milne-simpson method. Sci Hum, pp 61

[41] Biswas S, Roy TK (2018). Generalization of seikkala derivative and differential transform method for fuzzy volterra integro-differential equations. J Intell Fuzzy Syst.

[42] Biswas S, Roy TK (2018). Adomian decomposition method for solving initial value problem for fuzzy integro-differential equation with an application in volterra’s population model. J Fuzzy Math.

[43] Biswas S, Roy TK (2019). A semianalytical method for fuzzy integro-differential equations under generalized seikkala derivative. Soft Comput.

[44] Moi S, Biswas Suvankar, Pal(Sarkar) Smita (2020) Neutrosophic linear differential equation with a new concept of neutrosophic derivative. In: Neutrosophic Operational Research. Springer Nature. 10.1007/978-3-030-57197-9_21

[45] Deli I, Şubaş Y (2017). A ranking method of single valued neutrosophic numbers and its applications to multi-attribute decision making problems. Int J Mach Learn Cybern.

[46] Broumi S, Smarandache F (2015). Extended hausdorff distance and similarity measures for neutrosophic refined sets and their application in medical diagnosis. J New Theory.

[47] Sahin M, Deli I, Ulucay V (2017) Extension principle based on neutrosophic multi-fuzzy sets and algebraic operations. Infinite Study

